# Vitamin D Status and Mortality: A Systematic Review of Observational Studies

**DOI:** 10.3390/ijerph16030383

**Published:** 2019-01-29

**Authors:** Alicia K Heath, Iris Y Kim, Allison M Hodge, Dallas R English, David C Muller

**Affiliations:** 1 School of Public Health, Imperial College London, London W2 1PG, UK; david.muller@imperial.ac.uk; 2 Department of Epidemiology, Harvard T.H. Chan School of Public Health, Boston, MA 02115, USA; iris.kim@ndph.ox.ac.uk; 3 Nuffield Department of Population Health, University of Oxford, Oxford OX3 7LF, UK; 4 Centre for Epidemiology and Biostatistics, Melbourne School of Population and Global Health, University of Melbourne, Melbourne, Victoria 3010, Australia; Allison.Hodge@cancervic.org.au (A.M.H.); d.english@unimelb.edu.au (D.R.E.); 5 Cancer Epidemiology & Intelligence Division, Cancer Council Victoria, Melbourne, Victoria 3004, Australia

**Keywords:** vitamin D, vitamin D status, 25-hydroxyvitamin D, mortality, all-cause mortality, cause-specific mortality, cancer mortality, cardiovascular mortality, respiratory disease mortality, systematic review

## Abstract

Epidemiological evidence suggests that vitamin D deficiency is associated with increased mortality, but it is unclear whether this is explained by reverse causation, and if there are specific causes of death for which vitamin D might be important. We conducted a systematic review of observational studies investigating associations between circulating 25-hydroxyvitamin D (25(OH)D) concentration and all-cause or cause-specific mortality in generally healthy populations. Relevant studies were identified using PubMed and EMBASE searches. After screening 722 unique records and removing those that were ineligible, 84 articles were included in this review. The vast majority of studies reported inverse associations between 25(OH)D concentration and all-cause mortality. This association appeared to be non-linear, with progressively lower mortality with increasing 25(OH)D up to a point, beyond which there was no further decrease. There is moderate evidence that vitamin D status is inversely associated with cancer mortality and death due to respiratory diseases, while for cardiovascular mortality, there is weak evidence of an association in observational studies, which is not supported by the data from intervention or Mendelian randomization studies. The relationship between vitamin D status and other causes of death remains uncertain due to limited data. Larger long-term studies are required to clarify these associations.

## 1. Introduction

Evidence from observational studies suggests that vitamin D deficiency is associated with all-cause mortality [1,2,3,4]. However, it is unclear whether vitamin D is beneficial for overall health and longevity, or if it is a consequence or marker of poor health [5,6]. In addition, if vitamin D does confer a survival benefit, it is unknown whether there are specific causes of death for which vitamin D might have a protective role. 

An association between vitamin D status and mortality is biologically plausible. The vitamin D receptor and 25-hydroxyvitamin D-1α-hydroxylase enzyme, which produces the active vitamin D metabolite, 1,25-dihydroxyvitamin D (1,25(OH)_2_D), are widely expressed in tissues throughout the body [7,8]. In addition, vitamin D has anti-inflammatory, antiproliferative, prodifferentiative, antioxidative, and immunomodulatory effects, which might contribute to improved longevity [8,9]. 

Several systematic reviews and meta-analyses summarizing studies that examined the association between vitamin D status, assessed by circulating 25-hydroxyvitamin D (25(OH)D) concentration, and all-cause and/or cause-specific mortality have been published [1,2,3,4,10,11,12,13,14]. The meta-analyses are currently constrained by significant assay and laboratory differences in 25(OH)D measurements and a lack of standardization of methods [15,16]. Thus, caution is required when comparing 25(OH)D concentrations from studies with unstandardized 25(OH)D results, and some researchers have recommended that any further meta-analyses are deferred until better assay standardization is achieved [15]. Therefore, we conducted a systematic review and narrative synthesis of results without generating new summary measures of association.

This systematic review provides a comprehensive overview of observational data on vitamin D status and mortality in generally healthy populations, collating all of the published results to identify consistencies and inconsistencies in the literature and determine whether there is evidence of an association for specific causes of death.

## 2. Methods 

### 2.1. Search Strategy

We searched PubMed and EMBASE to identify relevant studies published from inception to 8 August 2018 (date of last search). The search strategy combined the following search terms related to vitamin D status and mortality and the appropriate study design, without language restriction: (vitamin D deficiency OR vitamin D insufficiency OR vitamin D status OR vitamin D metabolites OR 25-hydroxyvitamin D OR 25-hydroxy vitamin D OR 25(OH)D OR 25OHD) AND (mortality OR death) AND (cohort studies OR prospective studies OR follow-up study OR cohort OR prospective OR prospectively OR observational OR survey). The search was limited to humans and adults (the full search strategy can be viewed in the Supplementary Material).

### 2.2. Study Selection

Studies were eligible for inclusion if they assessed the association between measured circulating 25(OH)D concentration and all-cause mortality or cause-specific mortality in adults, and comprised participants from general populations, i.e. who were not selected based on pre-existing disease or being a patient for any particular condition. Studies involving residents of nursing homes or aged-care facilities were included if the participants were not selected on the basis of a disease.

Two reviewers (A.K.H. and I.Y.K.) independently screened the titles and abstracts of records identified from the database searches. Full text publications that met the selection criteria were retrieved and screened for eligibility. Discrepancies were resolved by consensus. Conference proceedings and duplicate publications of data from the same study population (using identical 25(OH)D comparisons but with fewer cases) were excluded. For completeness, multiple publications from the same study cohort were included as long as they provided estimates based on different 25(OH)D contrasts/metrics, or different causes of death. Studies that classified participants according to the presence/absence of a disease or condition such as frailty status, without showing results for all of the participants together, were excluded. Studies were ineligible if they did not provide sufficient data, including the 25(OH)D contrast or metric and relative risk estimates with corresponding 95% confidence intervals. Reference lists of relevant systematic reviews and meta-analyses and all of the selected articles were manually searched to identify additional relevant studies. 

### 2.3. Data Extraction

Two investigators independently extracted data using a data abstraction form, including information on: study name, study location, first author, publication year, causes of death investigated, age of participants at baseline, proportion of male/female participants, assay method, mean/median 25(OH)D concentration, duration of follow-up, number of participants, number of deaths, adjustment variables, 25(OH)D contrast or metric, effect estimates (hazard ratio (HR), relative risk (RR), odds ratio (OR), or mortality rate ratio) and corresponding 95% confidence intervals (CIs). If data were not presented, we performed calculations based on the information provided in the publication (where appropriate). Since some studies have reported higher mortality for very high 25(OH)D concentrations [17,18,19,20,21], where provided, data were also extracted for reported associations of high 25(OH)D versus levels in the middle of the concentration distribution. For consistency and ease of comparison across studies, 25(OH)D concentrations reported in ng/ml were converted to nmol/L by multiplying by 2.496. The figures show associations for low compared with high 25(OH)D or for a quantitative decrement in 25(OH)D (per 25 nmol/L decrement where data were available); data from publications that reported results using different metrics or for high versus low 25(OH)D or for an increment in 25(OH)D were converted to facilitate comparability. The original data extracted from publications are provided in the supplementary tables.

Since this was a qualitative synthesis, all of the published results involving a different 25(OH)D contrast or metric are included in the summary tables (see Supplementary Material), irrespective of subsequent or more comprehensive publications from the same study. However, to avoid duplication, only the most comprehensive results from each study (i.e. with the highest number of cases) are discussed and displayed in the figures.

## 3. Results

**Figure 1:**
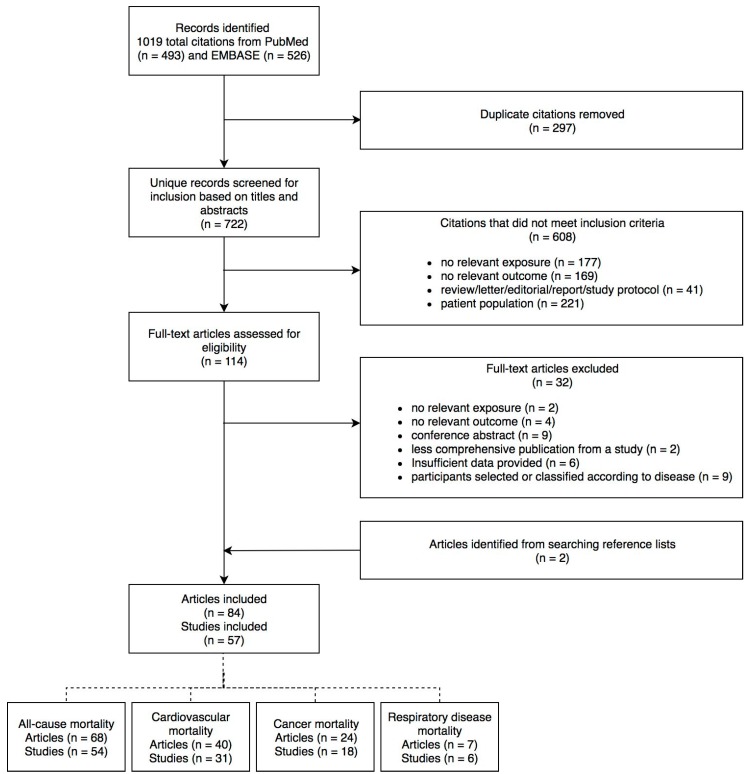
Flow diagram of selection of studies included in the review.

The systematic search yielded 493 records in PubMed and 526 records in EMBASE. After removing 297 duplicates, there were 722 records assessed for eligibility, of which 608 were excluded by screening titles and abstracts. After screening the full text of the remaining 114 articles, a further 32 were excluded, and two articles were identified from searching reference lists, resulting in a total of 84 articles reporting on 57 unique studies included in this review (Figure 1).

A full list of included articles, study characteristics, and adjusted confounders in the respective analyses is provided in Table S1. All of the articles were published between 2006–2018. The majority of the studies were in a community setting, except for three studies that recruited residents of nursing homes [22,23,24]. Almost all of the studies adjusted for important potential confounders, with the exceptions being the Octabaix study [25], Sao Paulo Ageing and Health study [26], and Yamato study [27], which reported unadjusted results. In the British National Diet and Nutrition Survey, adjustment was only made for age and sex [28]; a study among residents of a nursing home in Spain only adjusted for age, sex, and cystatin C [23]; and results from the Copenhagen vitamin D study (CopD study) were only adjusted for age, sex, and season [17,18]. The included studies comprised predominantly middle-aged or elderly participants, and follow-up time ranged from 1.7 to 37 years.

### 3.1. All-Cause Mortality

**Figure 2:**
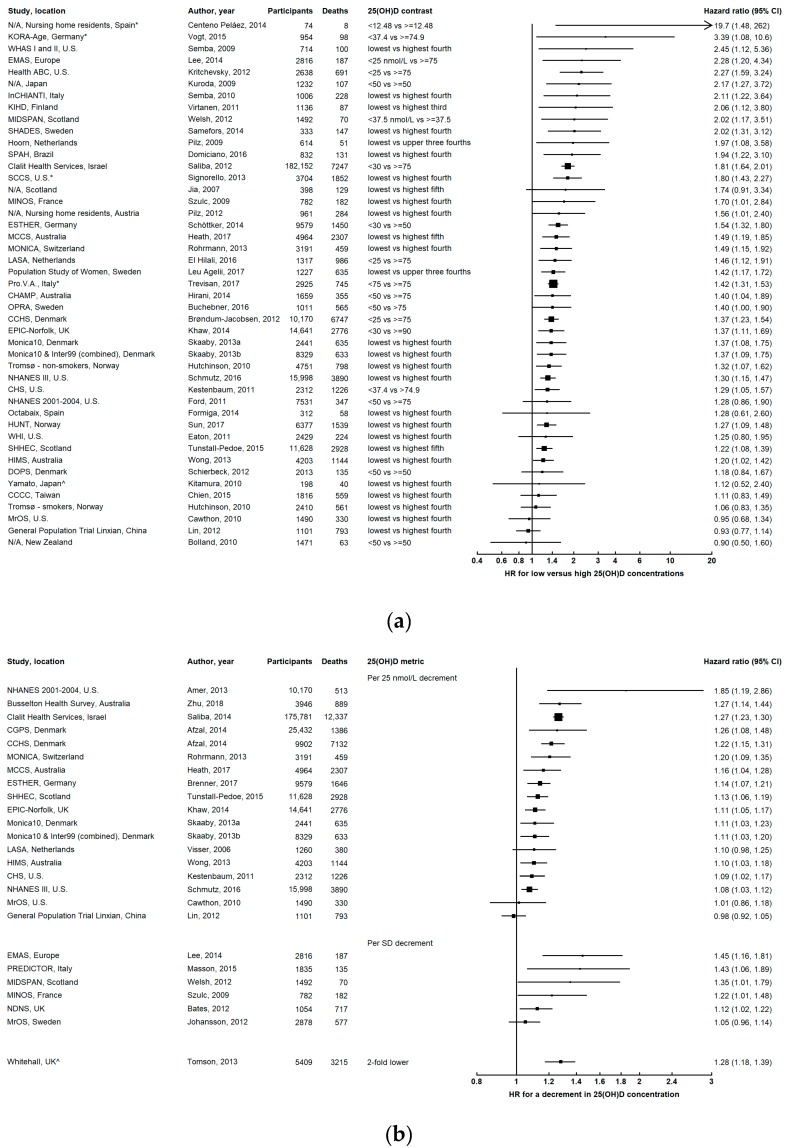

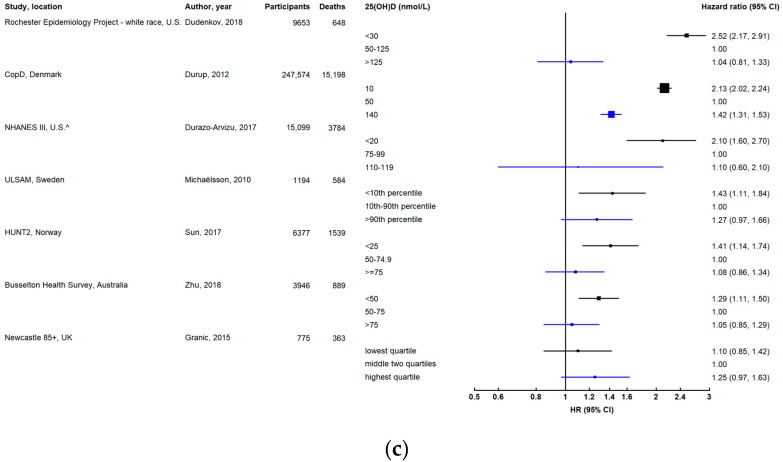
Results from prospective studies investigating all-cause mortality in relation to 25-hydroxyvitamin D (25(OH)D) concentration: (**a**) comparing low versus high concentrations categorically; (**b**) continuously, for a decrement in 25(OH)D concentration; (**c**) low (black) and high (blue) concentrations compared with levels in the middle of the 25(OH)D distribution. The squares represent hazard ratios, and horizontal lines are the 95% confidence intervals. Studies that estimated odds ratios or relative risks are denoted by * and ^, respectively. Axes are scaled differently in each panel to maximize the visualization of effect estimates.

Most studies reported inverse associations between 25(OH)D concentration and all-cause mortality (i.e. higher mortality for lower 25(OH)D) (Figure 2 and Table S2) [6,10,11,22,23,24,26,28,29,30,31,32,33,34,35,36,37,38,39,40,41,42,43,44,45,46,47,48,49,50,51,52,53,54,55,56,57,58,59,60,61]. These studies generally had long follow-up times, substantial numbers of participants and deaths, extensive adjustment for potential confounders, and encompassed broad geographical regions including North America, South America, Europe, Asia, and Australia, although the vast majority of studies were conducted in Europe and the United States (U.S.). Four other studies found associations of comparable magnitude, but estimates were accompanied by considerable uncertainty [25,62,63,64].

Reverse causation is a possible explanation for the observed increased mortality associated with low 25(OH)D concentrations, because people in poor health might be more likely to have vitamin D deficiency due to low sun exposure (and therefore less cutaneous synthesis of vitamin D_3_) and lower dietary intakes of vitamin D. In an attempt to mitigate this possibility, many of these studies controlled for prevalent chronic diseases such as cancer, cardiovascular disease (CVD), and/or diabetes at recruitment (Table S1), although whether this is appropriate remains contentious, given that these comorbidities may be on the causal pathway between vitamin D deficiency and mortality. Several studies excluded participants with prior cancer and/or CVD at baseline, or performed separate analyses for participants with and without specific chronic diseases, and found similar inverse associations (data not shown). In addition, vitamin D deficiency was associated with increased all-cause mortality among those who reported being in good/very good/excellent health in the ESTHER study (HR for 25(OH)D ˂ 30 compared with ≥50 nmol/L = 1.57, 95% CI 1.27–1.95) [6], and the Melbourne Collaborative Cohort Study (in which self-reported health status was assessed four years after baseline blood sample collection; HR per 25 nmol/L decrement = 1.18, 95% CI 1.02–1.37) [33].

Relatively few studies (n = 7) did not find an association between low 25(OH)D and all-cause mortality [19,27,65,66,67,68,69]. These studies were relatively small (fewer than 800 deaths), and all except the studies in Taiwan [67] and China [69] comprised elderly participants (mean age ≥74). In the Tromsø study, lower 25(OH)D concentrations were associated with higher all-cause mortality for non-smokers, but not for smokers [70].

A minority of studies (n = 3) reported higher mortality associated with very high 25(OH)D (Figure 2C) [17,19,20]. In the CopD study, increased all-cause mortality was observed for both low and high 25(OH)D, and the lowest risk was at 50–60 nmol/L [17]. However, this study utilized a general practice database, and was therefore unable to adjust for confounders other than age, sex, and season, and the median follow-up was only three years. In addition, relatively few participants (1.0%) had 25(OH)D concentrations >140 nmol/L, (versus 8.3% with 25(OH)D ˂ 10 nmol/L) [17]. In the Uppsala Longitudinal Study of Adult Men, there was a U-shaped association, with higher mortality for the lowest 10% (<46 nmol/L, HR = 1.43, 95% CI 1.11–1.84) and the highest 10% of 25(OH)D (>93 nmol/L, HR = 1.27, 95% CI 0.97–1.66) versus the 10^th^–90^th^ percentiles [20]. There was also a U-shaped relationship in the Newcastle 85+ study, but the increased risk for high 25(OH)D was only evident for women (RR for 25(OH)D ≥ 75 versus 25–74 nmol/L = 1.72, 95% CI 1.19–2.51) [19]. Further, this relationship differed depending on how 25(OH)D was categorized, and seemed to be mainly ascribed to women taking vitamin D supplements. When categorized as season-specific quartiles, the increased mortality risk (RR = 1.51, 95% CI 1.06–2.14) observed for the highest 25% versus the middle 50% of 25(OH)D was attenuated when restricted to women not taking vitamin D-containing supplements/prescribed medication (RR = 1.32, 95% CI 0.76–2.28). Using predefined cut-offs of >50 compared with 30–50 nmol/L, the corresponding HRs were 1.42 (95% CI 0.98–2.06) and 1.09 (95% CI 0.64–1.87), respectively. For men, neither low nor high 25(OH)D was associated with mortality [19]. Vitamin D supplementation is more likely to be recommended to elderly women than men [71]. A possible explanation for the U-shaped association in some studies could be that people with very high 25(OH)D were taking vitamin D supplements due to poor health, leading to a spurious association between high 25(OH)D concentrations and mortality [71]. Another possible explanation is that people with very high 25(OH)D concentrations have lower concentrations of the active vitamin D metabolite 1,25(OH)_2_D [72], which might in itself be associated with mortality [73,74]. Therefore, the results from these studies should be interpreted with caution.

Initial analyses of data from the Third National Health and Nutrition Examination Survey (NHANES III) suggested a “reverse J-shaped” association between 25(OH)D and all-cause mortality (albeit based on sparse data at low and high extremes of the 25(OH)D distribution), with a strong inverse association below 40 nmol/L, and a weak increased risk above 120 nmol/L [21]. However, after the standardization of 25(OH)D measurements to the National Institute of Standards and Technology reference measurement procedures, the shape of the association was different, with no increased nor decreased mortality risk at high 25(OH)D concentrations (>100 nmol/L) [75]. Below 40 nmol/L, the all-cause mortality risk increased with decreasing 25(OH)D, while above 40 nmol/L, the risk appeared to plateau. The radioimmunoassay used in NHANES III seemed to overestimate 25(OH)D [15], particularly at high concentrations (above about 102 nmol/L) [75]. After standardization, few individuals had 25(OH)D ≥ 120 nmol/L, and the association at very high concentrations could not be estimated [75], highlighting the importance of scrutinizing assay validity when interpreting results. Other studies that have examined very high concentrations of 25(OH)D compared with levels consistent with vitamin D sufficiency (generally around 50–75 nmol/L) have not found increased mortality for high 25(OH)D [50,57,76], and these findings are supported by meta-analyses [2,3,4].

#### 3.1.1. Dose-Response Relationship

Assessment of the shape of the dose-response relationship is constrained by few study participants with 25(OH)D concentrations at the extreme ends of the spectrum, and studies usually have insufficient statistical precision to accurately estimate the mortality risk for very high concentrations of 25(OH)D. Nevertheless, the shape that is emerging as the most consistent in the literature is a non-linear relationship, with decreasing all-cause mortality risk for increasing 25(OH)D below a certain threshold concentration, above which the risk plateaus, with no further reduction nor increase in risk at higher concentrations. However, there is considerable variability in the threshold 25(OH)D concentration reported across studies, reflecting a lack of assay standardization [15] and differences in vitamin D status across different populations and regions. In the Busselton Health Survey in Australia, there was a higher mortality risk below 65 nmol/L, with a plateau above 80 nmol/L [57]. In the Concord Health and Ageing in Men Project, a study of men aged ≥70 years in Australia, the optimum 25(OH)D range for mortality was 50–75 nmol/L, with substantially increased mortality at 25(OH)D < 50 nmol/L, and no further reduction in risk for 25(OH)D concentrations ≥75 nmol/L [34]. Similarly, in another study of Australian men, the Health in Men Study, there was an inverse association below 50 nmol/L, and a threshold at around 80 nmol/L [56]. In the ESTHER study in Germany, mortality increased with decreasing 25(OH)D below 75 nmol/L, above which the risk plateaued [6,30]. In the Nord-Trøndelag Health Study (HUNT Study) in Norway, there was an inverse association below 35 nmol/L, and the lowest risk was at 25(OH)D concentrations between 60–100 nmol/L (where the association remained constant) [50]. The NHANES 2001–2004 data also revealed a non-linear relationship, with a threshold of 52 nmol/L [61]. For white participants in the Rochester Epidemiology Project database, the threshold concentration was 75 nmol/L [76]. For both African Americans and non-African Americans in the Southern Community Cohort Study, the lowest mortality risk was observed at 25(OH)D around 87.5 nmol/L, above which the association plateaued [47]. In the Osteoporotic Fractures in Men Study (MrOS) in Sweden, despite a lack of association overall, there was a non-linear relationship in the short-term (<seven years after baseline), with higher mortality below 60 nmol/L and no association at higher 25(OH)D concentrations [68]. The 25(OH)D concentration with the lowest mortality risk in the Clalit Health Services cohort in Israel differed according to body mass index (BMI), which was 73.0 nmol/L for BMI < 25 kg/m^2^, 68.0 nmol/L for BMI 25–29.9 kg/m^2^, and 66.5 for BMI ≥ 30 kg/m^2^; there was no decreased nor increased risk for higher 25(OH)D concentrations [45]. No threshold or evidence against a linear dose-response relationship was found in the European Prospective Investigation into Cancer and Nutrition (EPIC)–Norfolk study [36], Melbourne Collaborative Cohort Study [33], MIDSPAN Family Study [55], MONICA study [43], or Scottish Heart Health Extended Cohort [52].

Meta-analyses have also found evidence of a non-linear dose-response relationship, with one revealing a plateau in the association for 25(OH)D above 90 nmol/L [2]. An individual participant data meta-analysis of standardized 25(OH)D found the lowest mortality risk at around 78 nmol/L, but risk was relatively constant between 50–125 nmol/L [3].

In light of a possible level of circulating 25(OH)D above which there is no further reduction in mortality risk, vitamin D supplementation is likely to confer a benefit only when 25(OH)D concentrations are low [77]. Brenner et al. (2017) used data from the ESTHER cohort study to estimate the expected reduction in mortality associated with incremental increases in 25(OH)D concentration, and demonstrated that the impact would be greater in individuals with vitamin D deficiency [30]. For example, the expected reduction in all-cause mortality for a 20 nmol/L increase in 25(OH)D was 34% for those with vitamin D deficiency versus 10% for all individuals [30]. Similarly, in the Health, Aging and Body Composition (Health ABC) Study, the predicted reduction in mortality for a given 25(OH)D increment was greater when the starting 25(OH)D concentration was lower; for example, the HR per 14 nmol/L increment was 0.81 (95% CI 0.73–0.89) for an initial 25(OH)D concentration of 25 nmol/L, and 0.94 (95% CI 0.89–0.99) for 75 nmol/L [37]. In an analysis of NHANES 2001–2004 data, there was a strong inverse association at low 25(OH)D concentrations (≤52 nmol/L, HR per 25 nmol/L increment = 0.54, 95% CI 0.35–0.84), but not at higher concentrations (>52 nmol/L, HR = 0.83, 95% CI 0.63–1.11) [61].

#### 3.1.2. Effect of Follow-Up Time and Repeated Measurements of 25(OH)D

A common limitation of cohort studies is having only one 25(OH)D measurement, which might not reflect long-term vitamin D status and the situation when the condition developed [78]. While this could be due to poor health causing vitamin D deficiency, vitamin D status is also influenced by factors other than health status, particularly sunlight exposure, season, and supplementation, and to a lesser extent diet, which might result in the loss of the predictive ability of a single 25(OH)D measurement with a long follow-up time. In the MINOS study of elderly men in France, 25(OH)D was associated with mortality mainly in the first three years of follow-up [51]. Similarly, in the MrOS study in Sweden, 25(OH)D predicted death most strongly approximately three years after baseline, beyond which the association was attenuated [68]. Leu-Agelii et al. (2017) examined whether the duration of follow-up affected the association between 25(OH)D and all-cause mortality in the Population Study of Women in Gothenburg with more than 30 years of follow-up. The association remained, but was attenuated with longer follow-up time (HRs at 17 years and 37 years for the lowest versus upper three 25(OH)D fourths = 1.96, 95% CI 1.25–3.08 and 1.42, 95% CI 1.17–1.72, respectively) [40]. 

The Malmö Osteoporosis Prospective Risk Assessment (OPRA) study in Sweden included repeat measurements of 25(OH)D, and was therefore able to assess the association of chronically low 25(OH)D concentrations (versus consistently high 25(OH)D) with mortality, and found an increased mortality risk over 10 years, even after adjusting for comorbidities (CVD, respiratory disease, kidney disease, diabetes mellitus, and osteoporosis) (HR for <50 versus >75 nmol/L = 1.8, 95% CI 1.1–2.8) [31]. In the InCHIANTI study, the mortality rate was lower for participants who had 25(OH)D concentrations above the median at both recruitment and the three-year follow-up than for those who had 25(OH)D concentrations below the median at both time points, or those who had 25(OH)D above the median at baseline, but dropped below the median at the three-year follow-up [46]. In an analysis of the ESTHER cohort, changes in vitamin D status during follow-up were taken into account by using repeated measurements of 25(OH)D and covariates, including self-rated health and frailty. When these variables were modeled time-dependently, the association between 25(OH)D and all-cause mortality (HR for 25(OH)D < 30 compared with ≥50 nmol/L = 1.60, 95% CI 1.37–1.86) was similar to that found in the analysis without time-dependent adjustment (HR = 1.54, 95% CI 1.32–1.80) [6], suggesting that a single measurement at baseline provided an adequate measure of longer-term vitamin D status.

#### 3.1.3. Evidence from Other Studies

The Vitamin D and Omega-3 Trial (VITAL) involving 25,871 healthy men and women in the U.S. did not find any benefit of vitamin D_3_ supplementation for all-cause mortality during a median follow-up of 5.3 years (HR = 0.99, 95% CI 0.87–1.12) [79]. The authors noted that extended follow-up may be required to detect an association, if it exists. However, the mean 25(OH)D concentration at baseline was 77 nmol/L, and only 12.7% of participants had 25(OH)D < 50 nmol/L [79]. As discussed above, vitamin D supplementation is likely to provide beneficial effects only for those with low 25(OH)D concentrations. Other randomized controlled trials (RCTs) have found a beneficial effect of vitamin D for all-cause mortality [1,5,80], although the magnitude of benefit in trials has been much smaller than expected, based on results from observational studies [5]. However, relatively small differences in 25(OH)D concentrations are achieved in RCTs, whereas a wider distribution of 25(OH)D concentrations exists in general populations [5]. In addition, the existing RCTs of vitamin D supplementation have been of relatively short duration and usually included vitamin D-replete individuals who may not experience any benefit. 

Findings from observational studies are supported by a Mendelian randomization study that showed that genetically-determined low 25(OH)D was associated with an increased risk of all-cause mortality that was of similar magnitude to that observed for measured plasma 25(OH)D (OR for genetically-determined 25 nmol/L lower 25(OH)D = 1.39, 95% CI 1.06–1.81 with corresponding observational OR = 1.27, 95% CI 1.14–1.40) [29].

#### 3.1.4. Vitamin D_2_ and Vitamin D_3_

A close inspection of trial data indicates that it might be important to consider the two different forms of vitamin D separately. Meta-analyses of RCTs have reported that vitamin D_3_ supplementation led to a small reduction in mortality (risk ratio = 0.94, 95% CI 0.91–0.98 in a Cochrane review meta-analysis [80] and RR = 0.89, 95% CI 0.80–0.99 in another meta-analysis [1]), while vitamin D_2_ supplementation had no effect or led to a marginal increase in mortality (risk ratio = 1.02, 95% CI 0.96–1.08 [80] and RR = 1.04, 95% CI 0.97–1.11 [1]). It is difficult to separate the effect of the type of vitamin D from the quality of the study; vitamin D_3_ trials were generally larger and better quality. By convention, observational studies have examined total 25(OH)D without assessing associations of 25(OH)D_2_ and 25(OH)D_3_ separately, as most assays did not distinguish between the two forms of vitamin D. In the Melbourne Collaborative Cohort Study, 25(OH)D_2_ and 25(OH)D_3_ were separately measured, and associations with all cause-mortality assessed. There was an inverse association between 25(OH)D_3_ and all-cause mortality (HR per 25 nmol/L increment in 25(OH)D_3_ = 0.85, 95% CI 0.77–0.95), whereas circulating 25(OH)D_2_ was associated with an increased risk (HR for detectable 25(OH)D_2_ = 1.80, 95% CI 1.09–2.97) [33]. In addition, total 25(OH)D was inversely associated with mortality among those with no detectable 25(OH)D_2_ (HR per 25 nmol/L increment in total 25(OH)D = 0.85, 95% CI 0.77–0.95), but not among those with detectable 25(OH)D_2_ (HR = 1.06, 95% CI 0.87–1.29) [33]. However, few participants had detectable circulating 25(OH)D_2_, and the results need to be verified in larger study populations with greater exposure to vitamin D_2_.

### 3.2. Cardiovascular Disease Mortality

**Figure 3:**
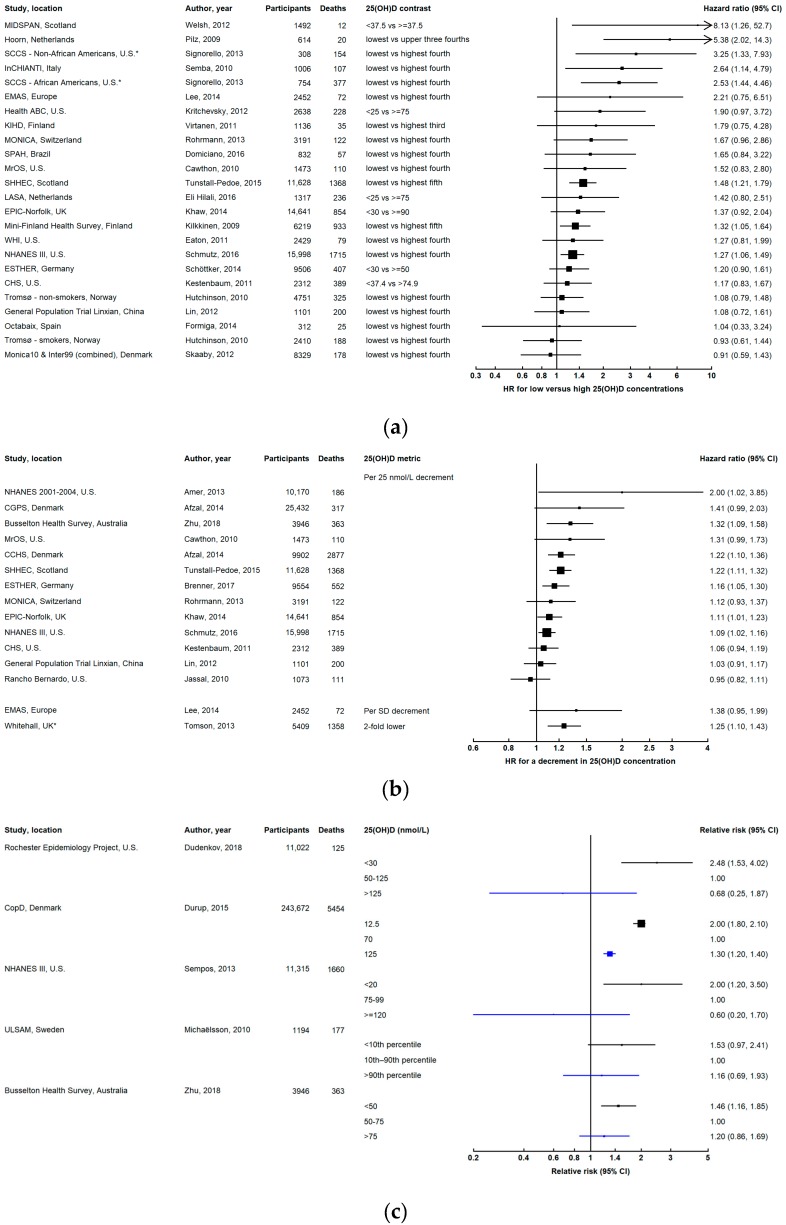
Results from prospective studies investigating cardiovascular mortality in relation to 25-hydroxyvitamin D (25(OH)D) concentration: (**a**) comparing low versus high concentrations categorically; (**b**) continuously, for a decrement in 25(OH)D concentration; (**c**) low (black) and high (blue) concentrations compared with levels in the middle of the 25(OH)D distribution. The squares represent hazard ratios and horizontal lines are the 95% confidence intervals. Studies that estimated odds ratios or relative risks are denoted by * and ^, respectively. Axes are scaled differently in each panel to maximize the visualization of effect estimates.

Low 25(OH)D concentrations were associated with an increased risk of death due to cardiovascular disease in the Busselton Health Survey [57], CopD study [18], Copenhagen City Heart Study [29], ESTHER study [30], EPIC–Norfolk study [36], Hoorn study [42], InCHIANTI study [46], MIDSPAN Family study [55], Mini-Finland Health Survey [81], NHANES III [58], NHANES 2001–2004 [61], Rochester Epidemiology Project [76], Southern Community Cohort Study [47], Scottish Heart Health Extended Cohort [52], and Whitehall study [11] (Figure 3). Several other studies found associations in the same direction, but estimates were accompanied by considerable uncertainty, as these studies had relatively few cardiovascular-related deaths [20,26,29,32,37,39,43,53,62,66]. No association was found in the Cardiovascular Health Study [35], General Population Trial of Linxian [69], Monica10 and Inter99 (combined) [82], Octabaix study [25], Rancho Bernardo study [83], or Tromsø study [70]. Lower 25(OH)D concentrations were associated with increased CVD mortality for women but not men in the MONICA study [43], while in the Scottish Heart Health Extended Cohort, the inverse association appeared to be stronger for men than women [52].

In the CopD study, similar to the findings for all-cause mortality, an increased risk of cardiovascular mortality (and death due to stroke and acute myocardial infarction) was reported for both very low and very high 25(OH)D (Figure 3C) [18]. However, other studies that examined the association for high 25(OH)D did not find increased CVD mortality for 25(OH)D at the upper end compared with the middle of the concentration distribution [21,76]. In the ESTHER study, the association was non-linear, with an increasing risk for decreasing 25(OH)D below 75 nmol/L, above which the risk plateaued [84]. Similarly, the risk leveled off at higher concentrations (above about 80 nmol/L) in the Busselton Health Survey, in which there was higher CVD mortality for 25(OH)D below 55 nmol/L [57].

#### 3.2.1. Subtypes of Cardiovascular Mortality

Associations of circulating 25(OH)D with specific subtypes of cardiovascular mortality are unclear due to limited data (Table S3). For death due to heart disease/myocardial infarction, all of the studies found an inverse association, which was particularly evident in the larger studies [10,29,85,86]. For death due to stroke, the results have been inconsistent, with low 25(OH)D strongly associated with increased risk only in the Mini-Finland Health Survey [81], CopD study [18], and for white participants in NHANES III [87].

#### 3.2.2. Evidence from Other Studies

In VITAL, supplementation with 2000 IU vitamin D_3_ per day did not lead to a lower rate of death from CVD (HR = 1.11, 95% CI 0.88–1.40) [79]. In addition, a Cochrane review meta-analysis of RCTs did not find a protective effect of vitamin D supplementation for CVD mortality (risk ratio = 0.98, 95% CI 0.90–1.07) [80]. Although several individual observational studies [11,29,30,36,42,46,47,52,55,57,58,61,76,81] and meta-analyses [1,3,11,13,14] have found an inverse association between 25(OH)D and cardiovascular mortality, a Mendelian randomization study reported the effect of genetically-determined 25(OH)D in the opposite direction. A lower risk of CVD mortality was associated with decreasing 25(OH)D (OR for genetically-determined 25 nmol/L lower 25(OH)D = 0.72, 95% CI 0.47–1.10), which contradicted the corresponding risk estimate for observational data (OR per 25 nmol/L lower 25(OH)D = 1.17, 95% CI 1.04–1.31) [29]. These findings raise the possibility that inverse associations between 25(OH)D concentration and cardiovascular mortality in observational studies could be due to confounding and reverse causation.

### 3.3. Cancer Mortality

**Figure 4:**
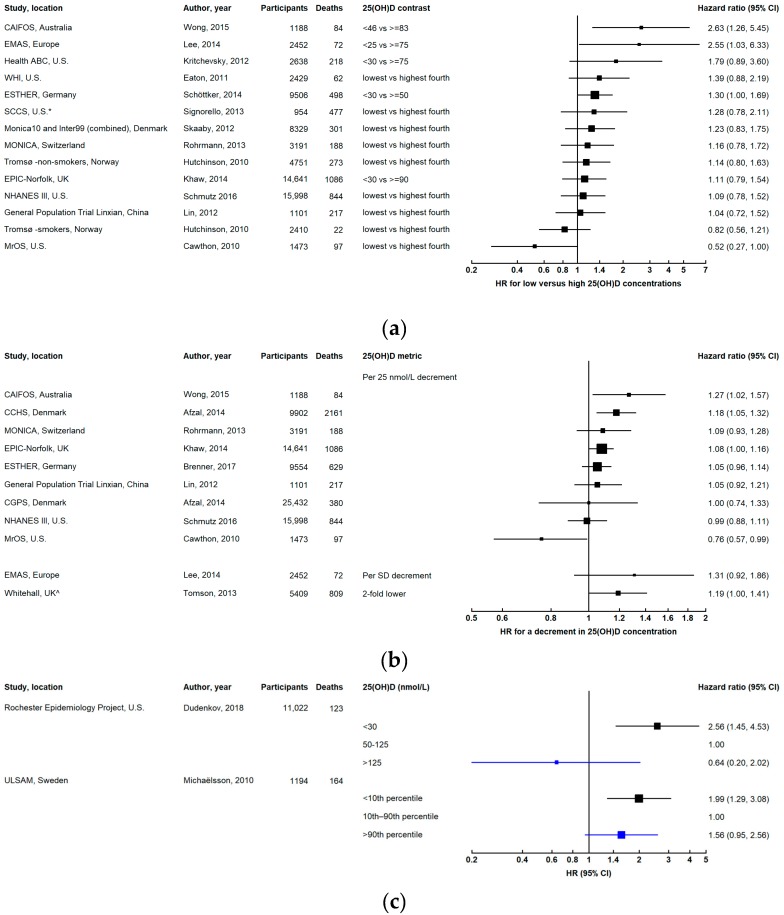
Results from prospective studies investigating cancer mortality in relation to 25-hydroxyvitamin D (25(OH)D) concentration: (**a**) comparing low versus high concentrations categorically; (**b**) continuously, for a decrement in 25(OH)D concentration; (**c**) low (black) and high (blue) concentrations compared with levels in the middle of the 25(OH)D distribution. The squares represent hazard ratios and horizontal lines are the 95% confidence intervals. Studies that estimated odds ratios or relative risks are denoted by * and ^, respectively. Axes are scaled differently in each panel to maximize the visualization of effect estimates.

This review only considers studies with cancer mortality as the outcome; however, it must be acknowledged that cancer mortality is determined by both cancer incidence and survival post-diagnosis, which may be differently associated with vitamin D. Studies investigating the association between 25(OH)D and cancer mortality have yielded inconsistent results, which may be related to population differences in the relative contribution of incidence and survival to cancer mortality, and the relative importance of different cancers that have different associations with vitamin D. Inverse associations were found in the Calcium Intake Fracture Outcome Study [88], Copenhagen City Heart Study [29], European Male Ageing Study [39], ESTHER study [84], Rochester Epidemiology Project [76], and Whitehall study [11], while there was no association in the Copenhagen General Population Study [29], General Population Trial of Linxian [89], MONICA study [43], NHANES III [58], or Trømso study [70] (Figure 4 and Table S4). In the EPIC-Norfolk study, there was a weak inverse association for continuous 25(OH)D, but little evidence of an association when comparing low versus high 25(OH)D categories [36]. The risk of cancer mortality for low 25(OH)D was increased for white but not black participants in the Health ABC study [37]. In the MONICA study, there was an inverse association for men, while the association was in the opposite direction for women, albeit with considerable uncertainty [43]. This is contrary to the sex-specific associations observed in the MONICA study for CVD mortality, in which there was an inverse association for women, but not men [43].

Results from the MrOS study in the U.S. suggested a decreased cancer mortality risk with lower 25(OH)D [66]. In an analysis of NHANES III data, higher cancer mortality risk was observed for men with circulating 25(OH)D ≥ 80 nmol/L compared with the lowest category (<37.5 nmol/L) [90]. These findings should be interpreted cautiously due to the aforementioned issues associated with unstandardized 25(OH)D data in NHANES III. There was no increased risk for very high 25(OH)D in another U.S. study [76] (Figure 4c).

A U-shaped association was reported in the Uppsala Longitudinal Study of Adult Men, with twofold higher mortality for the lowest and highest 5% of 25(OH)D [20]. In this study, U-shaped associations were particularly evident for death due to gastrointestinal cancers and enterohepatic cancers (Table S4) [20]. By contrast, in the ESTHER study, there was an inverse association below 75 nmol/L, leveling out at higher concentrations (consistent with the dose-response relationship for all-cause and cardiovascular mortality in the same study) [84].

#### 3.3.1. Site-Specific Cancer Mortality

The heterogeneous results for cancer mortality could reflect different associations for different cancer sites or types. However, data on the association of 25(OH)D with site-specific cancer mortality are sparse, with results from three studies for lung cancer, and only one or two studies for other cancers (Table S4). 

#### 3.3.2. Evidence from Other Studies

Meta-analyses have also yielded inconsistent results. In the largest meta-analysis of 25(OH)D and cancer mortality, the pooled RR for the lowest versus highest third of circulating 25(OH)D was 1.14 (95% CI 1.01–1.29) [1]. An individual participant data meta-analysis of standardized 25(OH)D in a European consortium found no association (RR for 25(OH)D < 30 versus 75–99.9 nmol/L = 1.10, 95% 0.75–1.61) [3], while another individual participant data meta-analysis found an association in those with prior cancer (RR for lowest versus highest fifth = 1.70, 95% CI 1.00–2.88), but not for those without prior cancer (RR = 1.03, 95% CI 0.89–1.20) [4], suggesting a benefit for cancer survival.

A Mendelian randomization analysis of United Kingdom (UK) Biobank data did not find any link between genetically-predicted 25(OH)D concentrations and cancer mortality [91]. In another Mendelian randomization study, genetically-determined lower 25(OH)D was associated with an increased risk of cancer mortality (OR for genetically-determined 25 nmol/L lower 25(OH)D = 1.56, 95% CI 1.03–2.36, with corresponding observational OR = 1.13, 95% CI 1.03–1.24) [29]. 

The results from two large RCTs have recently been published. In VITAL in the U.S., supplementation with 2000 IU vitamin D_3_ per day was associated with a 17% lower rate of death from cancer (HR = 0.83, 95% CI 0.67–1.02; excluding the first two years of follow-up HR = 0.75, 95% CI 0.59–0.96) [79]. In the Vitamin D Assessment (ViDA) study involving 5108 men and women in New Zealand, monthly high-dose (100,000 IU) vitamin D_3_ supplementation was not associated with decreased cancer mortality, but the null finding might be attributed to the dosing regimen (bolus rather than daily doses), short follow-up duration (median 3.3 years), insufficient cancer deaths (60 among those diagnosed with cancer after randomization), and small proportion of participants (25%) with vitamin D deficiency at baseline [92]. Meta-analyses of earlier RCTs support a beneficial role of vitamin D in cancer mortality. The Cochrane review meta-analysis reported a reduced risk of cancer mortality for vitamin D_3_ supplementation (risk ratio = 0.88, 95% CI 0.78–0.98) [80]. An identical effect estimate was found for vitamin D supplementation in another meta-analysis [93]. 

The overall evidence suggests that vitamin D might play a role in cancer mortality, but further research is required to ascertain whether there are benefits for specific cancers.

### 3.4. Respiratory Disease Mortality

**Figure 5:**
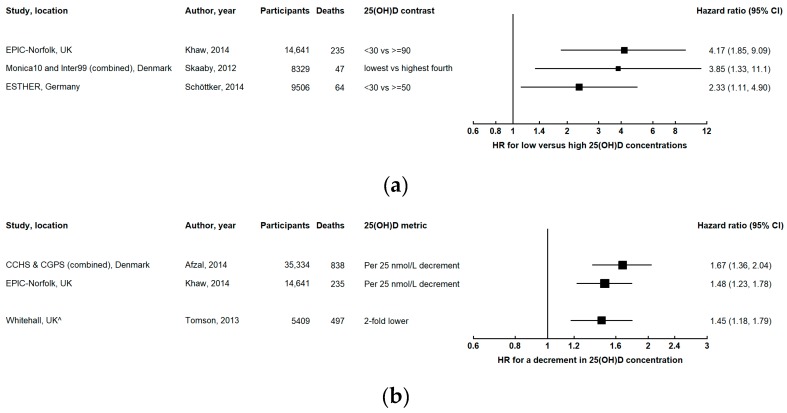

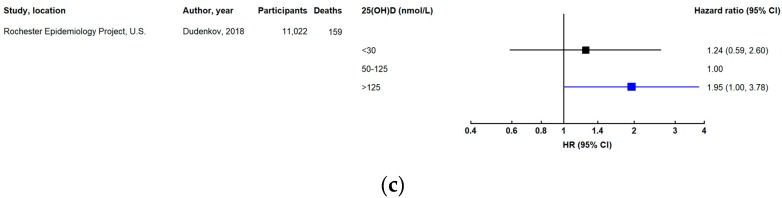
Results from prospective studies investigating respiratory disease mortality in relation to 25-hydroxyvitamin D (25(OH)D) concentration: (**a**) comparing low versus high concentrations categorically; (**b**) continuously, for a decrement in 25(OH)D concentration; (**c**) low (black) and high (blue) concentrations compared with levels in the middle of the 25(OH)D distribution. The squares represent hazard ratios and horizontal lines are the 95% confidence intervals. ^ Relative risk was estimated in the Whitehall study. Axes are scaled differently in each panel to maximize the visualization of effect estimates.

Six studies have investigated the association between 25(OH)D concentration and respiratory disease mortality. Five studies found an increased risk for low compared with high 25(OH)D concentrations (Figure 5 and Table S5) [6,11,29,36,82], while one study found an increased risk for very high (>125 nmol/L) compared with sufficient 25(OH)D levels (50–125 nmol/L) [76]. In the ESTHER study, the association was attenuated in time-dependent analyses accounting for repeat measurements of 25(OH)D and covariates [6]. In a meta-analysis combining results from three studies, the pooled RR for the lowest versus highest 25(OH)D third was 1.81 (95% CI 1.24–2.65) [1].

### 3.5. Other Causes of Death

Low vitamin D status was associated with a substantially increased risk of death due to diseases of the digestive system in both the ESTHER study [84] and Monica10 and Inter99 (combined) [82]. 

It is not possible to make inferences about the association between 25(OH)D concentrations and other causes of death, because results are currently only available from single studies for each of these causes (Table S6).

## 4. Discussion

Relatively consistent evidence exists for an inverse association between vitamin D status and all-cause mortality. This inverse association appears to be non-linear, with a progressively lower rate of death with increasing 25(OH)D up to a threshold concentration, above which risk remains constant. It is possible that any beneficial effects of vitamin D supplementation will only be conferred or be greatest in those who have very low 25(OH)D concentrations [77]. However, the identification of a specific 25(OH)D threshold is hampered by the lack of standardization of 25(OH)D measurements [15]. A possible explanation for the reverse J-shaped or U-shaped associations observed in a minority of studies is that very high 25(OH)D levels are due to vitamin D supplementation/vitamin D treatment in individuals with illness or poor health, and/or commencement of vitamin D supplementation only recently or later in life [71,94]. A major limitation of observational studies is that results could potentially be biased by reverse causation. Although it is likely that vitamin D status is influenced by health status, with poor health leading to vitamin D deficiency, ill health does not appear to fully account for the association between 25(OH)D concentration and all-cause mortality, since most of the studies that found strong evidence of an association adjusted for prior disease or excluded participants with certain chronic diseases at baseline. Lower 25(OH)D concentrations were also associated with increased all-cause mortality among participants who reported being in good to excellent health [6,33]. In addition, the association remains (but is slightly attenuated) with increasing follow-up durations, and persists after accounting for changes in 25(OH)D concentrations over time [6,31,40]. The separate associations of vitamins D_2_ and D_3_ with mortality requires further investigation, particularly considering findings from meta-analyses of RCTs, which have found reduced mortality for vitamin D_3_ supplementation, but no effect or even a potential increased risk for vitamin D_2_ supplementation [1,80].

For specific causes of death, there is moderate evidence that vitamin D status is inversely associated with cancer and respiratory disease mortality. However, there is currently insufficient data on site-specific cancer mortality to draw conclusions; therefore, it is unclear whether vitamin D could reduce mortality from all cancers or only cancers of specific sites. Tumor histology may be more pertinent than the anatomical site for any potential 25(OH)D and cancer relationship, and further investigation of associations by histological subtypes is warranted [95].

Although there is mounting evidence of an inverse association between 25(OH)D concentration and cancer mortality, investigations of cancer incidence have mostly yielded null results (except for colorectal cancer), suggesting that vitamin D might be involved in cancer progression rather than initial neoplastic processes [93,96,97]. A review by Jacobs et al. (2016) that summarized the evidence for the three main cancers investigated—colorectal, breast, and prostate—indicated that vitamin D may reduce colorectal cancer incidence and consequently mortality [98]. For breast cancer, on the other hand, the evidence suggested that the main impact was on progression rather than incidence, and via this mortality; meanwhile, for prostate cancer, the evidence was inconsistent, and was mostly related to progression and hence mortality [98]. Consistent with the impact of vitamin D on cancer mortality being due to post-diagnostic processes, in a meta-analysis of data from European and U.S. cohorts, circulating 25(OH)D was only associated with cancer mortality in participants who had already been diagnosed with cancer at baseline [4]. While survival in cancer patients was not the focus of this review, it is worth noting that results from several meta-analyses suggest a beneficial role of vitamin D in the progression and prognosis of various cancers; for example, higher 25(OH)D was associated with improved survival or reduced mortality in patients with colorectal cancer [99], prostate cancer [100], breast cancer [101,102], pancreatic cancer [103], and hematological malignancies [104].

There is inconsistent evidence for an association between vitamin D status and cardiovascular mortality, with the results from observational studies not supported by data from RCTs or Mendelian randomization. An insufficient number of studies have examined other causes of death to draw conclusions. 

The main limitations of observational studies are unmeasured or residual confounding, and the possibility of reverse causation. Therefore, they are not able to determine whether vitamin D is causally associated with mortality. Several large-scale long-term RCTs, which were designed to overcome some of the limitations of previous RCTs, were initiated over the last decade, and some results have recently been published [79,92,105,106,107,108]. A caveat is that these trials have included vitamin D-replete individuals, yet vitamin D supplementation trials assessing mortality are estimated to have much greater power when conducted among those with low 25(OH)D concentrations [30,77]. Therefore, it is important to take baseline vitamin D status into account where possible. In addition, current RCTs are unlikely to be large enough to examine specific causes of death (particularly rare causes); thus, further large long-term cohort studies are required to shed light on the association of vitamin D with less common causes of mortality.

## 5. Conclusions

There is strong evidence that vitamin D status is inversely associated with all-cause mortality. Vitamin D might be beneficial for cancer mortality and respiratory disease mortality, but further research is required to confirm any protective effect of vitamin D for these outcomes, and in particular, to investigate the relationship of vitamin D with specific cancer subtypes. Associations with other causes of death are inconclusive due to inconsistencies in the literature and sparse data. Results from further studies and ongoing RCTs are awaited to clarify these associations.

## Supplementary Materials

The following are available online at http://www.mdpi.com/1660-4601/16/3/383/s1, Table S1: Characteristics of prospective studies of 25-hydroxyvitamin D concentrations and mortality included in the review, Table S2: Results from prospective studies of circulating 25-hydroxyvitamin D and all-cause mortality, Table S3: Results from prospective studies of circulating 25-hydroxyvitamin D and cardiovascular mortality, Table S4: Results from prospective studies of circulating 25-hydroxyvitamin D and cancer mortality, Table S5: Results from prospective studies of circulating 25-hydroxyvitamin D and respiratory disease mortality, Table S6: Results from prospective studies of circulating 25-hydroxyvitamin D and other mortality, Search strategy.
