# Supplementary material for: Vitamin D Status and Mortality: A Systematic Review of Observational Studies
Source: Int J Environ Res Public Health. 2019 Jan 29;16(3):383. doi: 10.3390/ijerph16030383 (PMC6388383; doi:10.3390/ijerph16030383)
Supplement: Supplementary file 1 [file ijerph-16-00383-s001.pdf]

Vitamin D Status and Mortality: A Systematic Review of Observational Studies

Alicia K Heath, Iris Y Kim, Allison M Hodge, Dallas R English and David C Muller

SUPPLEMENTARY MATERIAL

Table S1: Characteristics of prospective studies of 25-hydroxyvitamin D concentrations and mortality included in the review.

First Author, Publication Year [Reference]	Study Name, Location	Mortality Outcomes	Mean Age (Years)	% Men	Vitamin D Assay	Mean 25(OH)D (nmol/L)	Follow-Up Time (years)	Adjustments
Bolland et al., 2010 [1]	N/A, New Zealand<br>*RCT of calcium supplementation in healthy postmenopausal women*	All-cause	74.0	0	RIA (DiaSorin)	50.9<br>(50.5 seasonally adjusted)	Mean 4.7	Season, treatment allocation (calcium or placebo), age, weight, smoking status, SBP, and history of ischaemic heart disease, stroke or transient ischaemic attack, dyslipidaemia, and diabetes
Centeno Peláez et al., 2014 [2]	N/A, Spain<br>*Nursing home residents*	All-cause	84	20.3	HPLC<br>*25(OH)D_3 only*	18.4	1.7	Age, sex, cystatin C
Jia et al., 2007 [3]	N/A, Scotland	All-cause	Median 80	52.0	RIA	Median 30.0	Median 5.8	Age, sex, taking five or more kinds of medicine, self-perceived health status, heart problem and/or diabetes at baseline., season of blood sampling, sunbathing, outdoor physical activity, use of a supplement containing vitamin D
Kuroda et al., 2009 [4]	N/A, Japan	All-cause	63.9	0	Competitive protein-binding assay	NR	Mean 6.9	Age, smoking status, alcohol, BMD, presence of cardiovascular events, dementia, malignancy
Pilz et al., 2012 [5]	N/A, Austria<br>*Nursing home residents*	All-cause	83.7	0	RIA (Immunodiagnostic Systems)	Median 17.5	Mean 2.3	Age, BMI, albumin, creatinine clearance, glycated haemoglobin, arterial hypertension, coronary artery disease, serum calcium, serum phosphate, PTH, knee extensor strength, and mobility status
Zhu et al., 2018 [6]	Busselton Health Survey, Australia	All-cause<br>CVD	52.5	42.8	Abbott ARCHITECT® 25-OH Vitamin D Assay (Abbott Laboratories)	Mean 60.6<br>Median 58.8	Max 20<br>(first 2 years of follow-up excluded)	age, sex, season of blood sampling, taking vitamin D supplements, CVD history, cholesterol, HDL cholesterol, smoking, diabetes, SBP, hypertension treatment, BMI, alcohol consumption, moderate and vigorous leisure time physical activity 150+ mins/week, log(glucose), log(triglyceride), log (CRP) and eGFR
Wong et al., 2015 [7]	Calcium Intake Fracture Outcome Study (CAIFOS), Australia<br>*RCT of calcium supplements to*	Cancer<br>Cancers of the digestive system<br>Lung cancer<br>Haematological cancers<br>Breast cancer	75.1	0	LC-MS/MS	Median 64	Median 10	Age, season, chronic kidney disease, smoking status

First Author, Publication Year [Reference]	Study Name, Location	Mortality Outcomes	Mean Age (Years)	% Men	Vitamin D Assay	Mean 25(OH)D (nmol/L)	Follow-Up Time (years)	Adjustments
	prevent osteoporotic fractures	Cancers of the central nervous system						
Chien et al., 2015 [8]	Chin-Shan Community Cardiovascular Cohort Study (CCCC), Taiwan	All-cause	60.2	55.0	ELISA	Median 50.6	Median 9.6	Age, sex, BMI, smoking status, alcohol consumption, marital status, education level, occupation, regular exercise, baseline hypertension, type 2 diabetes, LDL cholesterol and HDL cholesterol level
Brøndum-Jacobsen et al., 2012 [9]	Copenhagen City Heart Study (CCHS), Denmark	All-cause<br>Ischemic heart disease/MI	Median 57	44	CLIA (LIAISON® 25 OH Vitamin D TOTAL assay, DiaSorin)	Median 44.0	Median 21	Month of blood draw, sex, physical activity, smoking, diabetes mellitus, age, BMI, pack-years smoked, alcohol consumption, plasma total cholesterol, HDL cholesterol, SBP, and eGFR
Afzal et al., 2014 [10]	Copenhagen City Heart Study (CCHS), Denmark	All-cause<br>CVD<br>Cancer<br>Other (non-cancer/non-CVD)	Median 58	44	CLIA (LIAISON® 25 OH Vitamin D TOTAL assay, DiaSorin)	Median 41	Median 19.1 (max 32)	Age, sex, smoking status, cumulative tobacco consumption, alcohol consumption, leisure time physical activity, SBP, BMI, income, diabetes, plasma cholesterol, season (month and year of blood sample)
Afzal et al., 2014 [10]	Copenhagen General Population Study (CGPS), Denmark	All-cause<br>CVD<br>Cancer<br>Other (non-cancer/non-CVD)	Median 58	45	CLIA (LIAISON® 25 OH Vitamin D TOTAL assay, DiaSorin)	Median 52	Median 5.8 (max 9.4)	Age, sex, smoking status, cumulative tobacco consumption, alcohol consumption, leisure time physical activity, SBP, BMI, income, diabetes, plasma cholesterol, season (month and year of blood sample)
Afzal et al., 2014 [10]	Copenhagen City Heart Study (CCHS) and Copenhagen General Population Study (CGPS) (combined), Denmark	Coronary<br>Stroke<br>Lung cancer<br>Colorectal cancer<br>Respiratory	Median 58	45	CLIA (LIAISON® 25 OH Vitamin D TOTAL assay, DiaSorin)	Median 49	Median 19.1 (CCHS); 5.8 (CGPS)	Age, sex, smoking status, cumulative tobacco consumption, alcohol consumption, leisure time physical activity, SBP, BMI, income, diabetes, plasma cholesterol, season (month and year of blood sample), and study
Hirani et al., 2014 [11]	Concord Health and Ageing in Men Project (CHAMP), Australia	All-cause	77	100	RIA (DiaSorin)	55.9	Max 7	Age, season, BMI, physical activity, smoking status, self-rated general health, ≥4 comorbidities, and diabetes mellitus
Deo et al., 2011 [12]	Cardiovascular Health Study (CHS), U.S.	Sudden cardiac death	74	30.5	HPLC-MS/MS	NR	Median 14	Age, sex, race, season, clinic, education, physical activity, smoking, BMI, hypertension, diabetes and cystatin C-eGFR
Kestenbaum et al., 2011 [13]	Cardiovascular Health Study (CHS), U.S.	All-cause<br>CVD	74	30.3	HPLC-MS/MS	Mean 62.9	Median 14.0	Age, race, sex, season, clinic site, diabetes, antihypertensive medication, smoking status, education, physical activity, BMI, SBP, levels of CRP, total and HDL cholesterol, calcium,

First Author, Publication Year [Reference]	Study Name, Location	Mortality Outcomes	Mean Age (Years)	% Men	Vitamin D Assay	Mean 25(OH)D (nmol/L)	Follow-Up Time (years)	Adjustments
								phosphorus, PTH concentration, and cystatin C-eGFR
de Boer et al., 2012 [14]	Cardiovascular Health Study (CHS), U.S.	All-cause	74.0	30.3	HPLC-MS/MS	66.2	Median 11	Season, age, sex, clinical site, smoking status, BMI, and physical activity
Saliba et al., 2012 [15]	Clalit Health Services (CHS) cohort, Israel	All-cause	60.4	26.5	CLIA (LIAISON® 25 OH Vitamin D TOTAL assay, DiaSorin)	Median 49.4	Median 2.4	Age, sex, ethnicity, seasonality, use of vitamin D supplements, use of statins, histories of hypertension, diabetes mellitus, CVD, and cancer, smoking status, BMI, socioeconomic status, HDL cholesterol, LDL cholesterol, corrected serum calcium for albumin level, and GFR
Saliba et al., 2014 [16]	Clalit Health Services (CHS) cohort, Israel	All-cause	60.6	26.5	CLIA (LIAISON® 25 OH Vitamin D TOTAL assay, DiaSorin)	50.9	Median 4	Age, sex, ethnicity, season, vitamin D supplements, statins treatment, smoking status, and histories of hypertension, diabetes mellitus, CVD, and cancer
Durup et al., 2012 [17]	Copenhagen vitamin D (CopD) study, Denmark	All-cause	51.0	34.8	CLIA (LIAISON® 25 OH Vitamin D TOTAL assay, DiaSorin) and enzyme immunoassay (OCTEIA 25(OH)D_3 and 25(OH)D_2, Immunodiagnostic Systems)	NR	Median 3.1	Season of blood sampling, age, sex
Durup et al., 2015 [18]	Copenhagen vitamin D (CopD) study, Denmark	CVD<br>Stroke<br>Acute MI	51.3	34.7	CLIA (LIAISON® 25 OH Vitamin D TOTAL assay, DiaSorin) and enzyme immunoassay (OCTEIA 25(OH)D_3 and 25(OH)D_2, Immunodiagnostic Systems)	Mean 49.5	Median 3.1	Season of blood sampling, age, sex
Schierbeck et al., 2012 [19]	Danish Osteoporosis Prevention Study (DOPS), Denmark	All-cause	50	0	RIA	NR	Max 16	Age, smoking, SBP, family history of MI, education level, and hip-waist ratio

First Author, Publication Year [Reference]	Study Name, Location	Mortality Outcomes	Mean Age (Years)	% Men	Vitamin D Assay	Mean 25(OH)D (nmol/L)	Follow-Up Time (years)	Adjustments
Lee et al., 2014 [20]	European Male Ageing Study (EMAS), Europe [1]	All-cause<br>CVD<br>Cancer	60	100	RIA (DiaSorin)	Median 58.0	Median 4.3	Age, centre, smoking status, alcohol consumption, self-reported morbidities, PASE score, PPT rating, and serum creatinine
Khaw e al., 2014 [21]	EPIC-Norfolk, UK	All-cause<br>CVD<br>Cancer<br>Respiratory<br>Other (non-CVD/non-cancer/non-respiratory)	Range 42-82	44.3	LC-MS/MS	56.6	Mean 13	Age, sex, month of blood draw, BMI, cigarette smoking, physical activity, alcohol intake, plasma vitamin C, social class, education, diabetes, history of CVD, and history of cancer
Perna et al., 2013 [22]	ESTHER study, Germany	CVD<br>CHD<br>Stroke	Range 50-74	40.7	Immunoassays: DiaSorin-Liaison (women), IDS-iSYS (Immunodiagnostic Systems) (men); measurements standardised to LC-MS/MS	NR	Mean 9.2	Age, sex, season of blood draw, BMI, smoking status, physical activity, total cholesterol, CRP, family history of CVD, fish consumption, and regular multivitamin supplement intake
Schöttker et al., 2013 [23]	ESTHER study, Germany	All-cause<br>CVD<br>Cancer<br>Respiratory diseases	62	43.8	Immunoassays: DiaSorin-Liaison (women), IDS-iSYS (Immunodiagnostic Systems) (men); measurements standardised to LC-MS/MS	51.1	Median 9.5	Age, sex, season of blood draw, regular intake of multivitamin supplements, fish consumption, BMI, scholarly education, physical activity, smoking status, SBP, chronic kidney disease, log (serum CRP concentrations), and total cholesterol<br>Respiratory disease mortality was adjusted for age, sex, season, smoking, and physical activity
Schöttker et al., 2014 [24]	ESTHER study, Germany	All-cause<br>CVD<br>Cancer<br>Respiratory diseases<br>Diseases of the digestive system<br>Diabetes and obesity<br>Diseases of the genitourinary system<br>Diseases of the nervous system and mental disorders<br>Other (Infectious and not otherwise classified diseases)	62.2	43.8	Immunoassays: DiaSorin-Liaison (women), IDS-iSYS (Immunodiagnostic Systems) (men); measurements standardised to LC-MS/MS	NR	Median 11.5 (all-cause)<br>10.4 (cause-specific)	Age, sex, season of blood draw, education, BMI, smoking, light physical activity, self-rated health, frailty index and 25(OH)D<br>(repeated measurements of 25(OH)D and repeated measurements of covariates were used in time-dependent analyses)<br><br>Self-rated health was not adjusted for in analyses of death due to diseases of the genitourinary system, diseases of the nervous system and mental disorders, and other diseases

First Author, Publication Year [Reference]	Study Name, Location	Mortality Outcomes	Mean Age (Years)	% Men	Vitamin D Assay	Mean 25(OH)D (nmol/L)	Follow-Up Time (years)	Adjustments
Brenner et al., 2017 [25]	ESTHER study, Germany	All-cause<br>CVD<br>Cancer	62	43.8	Immunoassays: DiaSorin-Liaison (women), IDS-iSYS (Immunodiagnostic Systems) (men); measurements standardised to LC-MS/MS	51.1	Median 12.4	Age, sex, season of blood draw, regular intake of any multivitamin supplements, fish consumption, BMI, school education, physical activity, smoking status, SBP, chronic kidney disease, CRP, and total cholesterol.
Lin et al., 2012 [26]	General Population Trial of Linxian, China	All-cause<br>CVD<br>Cerebrovascular<br>Cancer<br>Upper gastrointestinal cancer	56.5	55	Enzyme immunoassay (OCTEIA 25-hydrocy vitamin D enzyme immunoassay, IDS)	Median 31.9	Median 12	Age, sex, tobacco smoking, alcohol consumption, BMI and hypertension
Kritchevsky et al., 2012 [27]	Health, Aging and Body Composition (Health ABC) Study, U.S.	All-cause<br>CVD<br>Cancer<br>Non-cancer/non-CVD	74.7	48.8	RIA (DiaSorin)	64.4	Median 7.8	Age, sex, race, education, season, field centre, smoking status, pack-years, alcohol consumption, BMI, time walking, usual 20-m walking speed, eGFR, cognition, depressive symptoms, IL-6, cholesterol, and prevalent diabetes, hypertension, CVD, cancer, or lung disease
Wong et al., 2013 [28]	Health in Men Study (HIMS), Australia	All-cause	76	100	CLIA (LIAISON® 25 OH Vitamin D TOTAL assay, DiaSorin)	68.3	Mean 6.7	Age, education, living circumstance, smoking, CVD, diabetes, hypertension, dyslipidaemia, Charlson's comorbidity index, BMI, renal function (eGFR), seasonality, and baseline frailty status
Pilz et al., 2009 [29]	Hoorn Study, the Netherlands	All-cause<br>CVD	Range 50-75	49.3	Competitive protein binding assay (DiaSorin)	Women 50.8<br>Men 56.5	Mean 6.2	Age, sex, diabetes mellitus, smoking status, arterial hypertension, HDL cholesterol, GFR, waist-to-hip ratio
Sun et al., 2017 [30]	Nord-Trøndelag Health Study 2 (HUNT2), Norway	All-cause	NR	46.8	CLIA (LIAISON® 25 OH Vitamin D TOTAL assay, DiaSorin)	Mean 47.3	Median 18.5	Season of blood draw, age, sex, BMI, smoking, alcohol consumption, physical activity, education, economic difficulties, chronic illness (MI/angina/stroke/ diabetes/cancer)
Semba et al., 2010 [31]	InCHIANTI study, Italy	All-cause<br>CVD	76<br>≥65	75.0	RIA (DiaSorin)	Median 39.9	Mean 6.5	Age, sex, education, season, BMI, smoking, aspirin use, physical activity, total cholesterol, HDL cholesterol, MMSE score, renal insufficiency, and (for all-cause mortality) hypertension, diabetes, heart failure, and stroke
Virtanen et al., 2011 [32]	Kuopio Ischaemic Heart Disease Risk Factor (KIHD) Study, Finland	All-cause<br>CVD	61.8	48.6	HPLC using diode array detector<br>*Results for 25(OH)D_3 reported*	Mean 43.7	Mean 9.1	Age, sex, examination year, examination month, diabetes, treated hypertension. BMI, smoking, education years, medication for hyperlipidaemia

First Author, Publication Year [Reference]	Study Name, Location	Mortality Outcomes	Mean Age (Years)	% Men	Vitamin D Assay	Mean 25(OH)D (nmol/L)	Follow-Up Time (years)	Adjustments
Mursu et al., 2015 [33]	Kuopio Ischaemic Heart Disease Risk Factor (KIHD) Study, Finland	Disease death (all-causes excluding deaths due to accidents or suicides)	52	100	HPLC using coulometric electrode array detection; *25(OH)D*$_3$ *only*	43.5	Mean 22.2	Age, month and year of blood collection, BMI, smoking, leisure-time physical activity, education years, income, marital status, serum long-chain omega-3 polyunsaturated fatty acids, and intakes of alcohol, energy, fruits, berries and vegetables
Vogt et al., 2015 [34]	Cooperative Health Research in the Region of Augsburg (KORA)-Age Study, Germany	All-cause	75.5	50.9	ECLIA (Roche)	46.7	Mean 2.9	Age, sex, season of blood collection, BMI, baseline frailty status, education, smoking status, alcohol consumption, CVD, diabetes mellitus, multimorbidity
Visser et al., 2006 [35]	Longitudinal Aging Study Amsterdam (LASA), the Netherlands	All-cause	≥65	48.8	Competitive protein-binding assay (Nichols Diagnostics)	Median 51.8	6	Age, sex, education, chronic diseases, serum creatinine concentration, cognitive status, depressive symptoms, BMI, smoking status, alcohol consumption, physical activity, and frailty indicators: mobility performance, low serum albumin concentration, and low serum total cholesterol concentration
El Hilali et al., 2016 [36]	Longitudinal Aging Study Amsterdam (LASA), the Netherlands	All-cause<br>CVD	Median 75	48.7	Competitive protein-binding assay (Nichols Diagnostics)	NR	Mean 18 (all-cause)<br>13 (CVD mortality)	Season, age, sex, education, smoking, alcohol consumption, BMI, physical activity, number of chronic diseases, eGFR
Heath et al., 2017 [37]	Melbourne Collaborative Cohort Study (MCCS), Australia	All-cause	57.1	55.8	LC-MS/MS	Median 48.4	Mean 13.7	Season, age, sex, country of birth, area-based index of socioeconomic disadvantage, educational attainment, total energy intake, Mediterranean diet score, alcohol intake, smoking status, physical activity, waist circumference, baseline diabetes mellitus, and history of hypertension, angina, MI, and stroke
Welsh et al., 2012 [38]	MIDSPAN Family Study, Scotland	All-cause<br>CVD<br>Non-CVD	45	44.2	LC-MS/MS	Median 46.4	Median 14.4	Age, sex, season, diabetes, glucose, smoking, SBP, total cholesterol, ,HDL cholesterol, BMI, triglycerides, waist circumference, creatinine, CRP, insulin, highest educational level, social class, deprivation category, percent fat from diet, alcohol intake, high and low fibre in diet, current mediation (ACE inhibitors, antihypertensives, aspirin, insulin, oral hypoglycaemics, sartans, and statins), baseline CHD, low baseline physical activity, percent predicted FEV1, vitamin D intake, PTH, and adjusted calcium

First Author, Publication Year [Reference]	Study Name, Location	Mortality Outcomes	Mean Age (Years)	% Men	Vitamin D Assay	Mean 25(OH)D (nmol/L)	Follow-Up Time (years)	Adjustments
Kilkkinen et al., 2009 [39]	Mini-Finland Health Survey, Finland	CVD<br>Cerebrovascular disease<br>Haemorrhagic stroke<br>Ischaemic stroke<br>Coronary disease	49.4	45.3	RIA (DiaSorin)	43.4	Median 27.1	Age, sex, marital status, educational level, BMI, alcohol consumption, smoking, leisure time physical activity, and season of baseline examination
Szulc et al., 2009 [40]	MINOS, France	All-cause	65<br>Range 50-85	100	RIA (DiaSorin)	NR	Max 10	Age, BMI, smoking, physical performance and activity, vitamin D supplementation, and health status
Rohrmann et al., 2013 [41]	MONICA, Switzerland	All-cause<br>CVD<br>Cancer	47.1	51.6	Protein-binding assay	NR	Mean 18.0	Sex, age, season, intake of fish and meat, nationality, SBP, and smoking status
Skaaby et al., 2013a [42]	Monica10, Denmark	All-cause	55.4	50.2	CLIA (IDS iSYS, Immunodiagnostic Systems)	Median 61.0	Median 17.0	Sex, education, season, diet, BMI, physical activity, smoking habits, alcohol intake, and levels of liver enzymes (alanine aminotransferase, aspartate aminotransferase, and gamma glutamyl transferase)
Skaaby et al., 2012 [43]	Monica10 and Inter99 (combined), Denmark	CVD<br>Cancer<br>Endocrine, nutritional and metabolic diseases<br>Mental and behavioural disorders<br>Diseases of the nervous system<br>Diseases of the respiratory system<br>Diseases of the digestive system	Median 50	49.5	CLIA (IDS iSYS, Immunodiagnostic Systems) (Monica10) and<br>HPLC (Inter99)	Median 61.0 (Monica10)<br>48.0 (Inter99)	Median 10	Age, study group, sex, education, season of blood sample, intake of fish, physical activity, smoking habits, BMI, and alcohol consumption
Skaaby et al., 2013b [44]	Monica10 and Inter99 (combined), Denmark	All-cause	55.4 (Monica10)<br>46.1 (Inter99)	50.2 (Monica 10)<br>49.2 (Inter99)	CLIA (IDS iSYS, Immunodiagnostic Systems) (Monica10) and<br>HPLC (Inter99)	Median 61.0 (Monica10)<br>48.0 (Inter99)	Mean 10.2	Age, study group, sex, education, season of blood sample, intake of fish, physical activity, smoking habits, BMI, and alcohol consumption
Cawthon et al., 2010 [45]	Osteoporotic Fractures in Men (MrOS) study, U.S.	All-cause<br>CVD<br>Cancer<br>Non-cancer/non-CVD	73.7	100	MS	Median 62.9	Mean 7.3	Age, clinical centre, season of blood draw, serum calcium and phosphate, GFR, percentage body fat, weight, race, health status, presence of at least one medical condition, alcohol use, education, activity level (PASE score), marital status, and presence of a functional or mobility limitation

First Author, Publication Year [Reference]	Study Name, Location	Mortality Outcomes	Mean Age (Years)	% Men	Vitamin D Assay	Mean 25(OH)D (nmol/L)	Follow-Up Time (years)	Adjustments
Johansson et al., 2012 [46]	Osteoporotic Fractures in Men Study (MrOS), Sweden	All-cause	75.5	100	RIA (DiaSorin)	Median 67	Mean 6.0	Age, time since baseline, general health, BMD, diabetes, outdoor activity, physical activity (walking)
Bates et al., 2012 [47]	National Diet and Nutrition Survey (NDNS) of People Aged 65 Years and Over, UK	All-cause	76.6	51.0	Competitive protein binding (DiaSorin)	Men 58.4<br>Women 49.6	Max 13-14	Age, sex
Granic et al., 2015 [48]	Newcastle 85+ Study, UK	All-cause	≥85	39.2	RIA (DiaSorin)	45.4	Mean 6	Season, sex, education, marital status, number of income sources, smoking, alcohol intake, physical activity, depressive symptoms, cognitive impairment, number of chronic diseases, renal impairment, and waist–hip ratio
Freedman et al., 2007 [49]	NHANES III, U.S. 1988-1994<br>Follow-up to 2000	Cancer<br>Colorectal cancer<br>Oesophageal, stomach, liver and pancreatic cancer<br>Lung cancer<br>Breast cancer<br>Prostate cancer<br>Non-Hodgkin lymphoma/leukaemia<br>Other cancers (including buccal cavity, larynx, melanoma, gynaecologic sites, kidney, bladder, brain, multiple myeloma, and others)	44.0	45.4	RIA (DiaSorin)	65.6	Median 8.9	Age, race/ethnicity, sex, and smoking history (pack-years)
Melamed et al., 2008 [50]	NHANES III, U.S. 1988-1994<br>Follow-up to 2000	All-cause<br>CVD<br>Cancer<br>Infectious diseases<br>External causes	44.8	45.5	RIA (DiaSorin)	Median 60.9	Median 8.7	Age, sex, race, season, smoking, serum albumin level, log CRP level, BMI, physical activity level, use of vitamin D supplementation, and low socioeconomic status, and (for all-cause, CVD and infectious disease mortality analyses) hypertension, history of CVD, diabetes, HDL cholesterol, total cholesterol, use of cholesterol-lowering medications, eGFR, and log albumin to creatinine ratio

First Author, Publication Year [Reference]	Study Name, Location	Mortality Outcomes	Mean Age (Years)	% Men	Vitamin D Assay	Mean 25(OH)D (nmol/L)	Follow-Up Time (years)	Adjustments
Ginde et al., 2009 [51]	NHANES III, U.S. 1988-1994 Follow-up to 2000	All-cause<br>CVD<br>Non-CVD	73	44	RIA (DiaSorin)	Median 66.0	Median 7.3	Season, age, sex, race/ethnicity, poverty:income ratio, region, BMI, physical activity, smoking status, cigarette pack years, asthma, COPD, renal function, hypertension, diabetes mellitus, hyperlipidaemia, history of MI, history of stroke, and history of cancer (non-skin)
Fiscella et al., 2010 [52]	NHANES III, U.S. 1988-1994 Follow-up to 2000	CVD	43.6	48	RIA (DiaSorin)	74.0	9.0	Age, log(age), sex, interview month, region, race and ethnicity, household income, smoking status, physical inactivity, self-rated health, self-reported diabetes, self-reported baseline CVD, BMI, SBP, eGFR, total cholesterol, serum albumin, CRP, urinary albumin-creatinine ratio, and chronic kidney disease
Liu et al., 2012 [53]	NHANES III, U.S. 1988-1994 Follow-up to 2000	CVD<br>Heart failure<br>Cancer<br>Premature all-cause (<75 years of age)	≥35	46.7	RIA (DiaSorin)	NR	Mean 8	Age, sex, race/ethnicity, marital status, education, smoking, alcohol consumption, physical activity, BMI, and diabetes
Freedman et al., 2010 [54]	NHANES III, U.S. 1998-1994 Follow-up to 2006	Cancer<br>Colorectal cancer<br>Oesophageal, stomach, liver and pancreatic cancer<br>Lung cancer<br>Breast cancer<br>Prostate cancer<br>Non-Hodgkin lymphoma/leukaemia<br>Other cancers (including buccal cavity, larynx, melanoma, gynaecologic sites, kidney, bladder, brain, multiple myeloma, and others)	44.0	47.0	RIA (DiaSorin)	NR	Mean 13.4	Age, sex, race/ethnicity, smoking history, and BMI
Fiscella et al., 2011 [55]	NHANES III, U.S. 1988-1994 Follow-up to 2006	Colorectal cancer	≥20	53.0	RIA (DiaSorin)	73.6	NR	Month, region, age, sex, race/ethnicity, educational level, health insurance, BMI, history of colorectal cancer at baseline
Cheng et al., 2012 [56]	NHANES III, U.S. 1988-1994 Follow-up to 2006	Lung cancer	43.7	45.5	RIA (DiaSorin)	Median 60.9	Max 18	Age, sex, race/ethnicity, smoking status, census region, BMI

First Author, Publication Year [Reference]	Study Name, Location	Mortality Outcomes	Mean Age (Years)	% Men	Vitamin D Assay	Mean 25(OH)D (nmol/L)	Follow-Up Time (years)	Adjustments
Michos et al., 2012 [57]	NHANES III, U.S. 1988-1994 Follow-up to 2006	Stroke	50.1 (whites), 46.6 (blacks)	45.8	RIA (DiaSorin)	Mean 76.9 (whites), 48.4 (blacks)	Median 14.1	Age, sex, season, income, education, BMI, smoking, physical activity, alcohol use, CRP, diabetes, hypertension, and hypercholesterolemia
Smit et al., 2012 [58]	NHANES III, U.S. 1988-1994 Follow-up to 2006	All-cause	70.8 ≥60	41.1	RIA (DiaSorin)	68.2	Median 12.6	Age, BMI, race/ethnicity, sex, smoking, education, chronic disease index, and latitude
Deng et al., 2013 [59]	NHANES III, U.S. Follow-up to 2006	All-cause, CVD Colorectal cancer	≥17	NR	RIA (DiaSorin)	NR	Max 18	Age, race, sex, BMI, education attainment, household income, smoking status, alcohol use, physical activity, month of blood collection, intakes of total energy, phosphorus, calcium and magnesium
Sempos et al., 2013 [60]	NHANES III, U.S. 1988-1994 Follow-up to 2006	All-cause CVD Cancer Other (non-CVD/non-cancer/non-accidents)	45	49	RIA (DiaSorin)	64	15	Age, sex, race/ethnicity, season, and (for all-cause mortality) self-reported diabetes, congestive heart failure, stroke, heart attack, cancer other than skin cancer, GFR, BMI, physical activity, current smoking, education, and medication use
Ford et al., 2015 [61]	NHANES III, U.S. 1988-1994 Follow-up to 2006	All-cause	20-79	48.8	RIA (DiaSorin)	74.5 Median 71.6	Mean 14.2 Median 14.5	Month, age, sex, race or ethnicity, education, smoking status, alcohol use, leisure-time physical activity, use of vitamin or mineral supplements, SBP, HDL cholesterol, non-HDL cholesterol, BMI, CRP, albumin–creatinine ratio, health status, diabetes, history of MI and history of stroke
Daraghmeh et al., 2016 [62]	NHANES III, U.S. 1988-1994 Follow-up to 2006	All-cause CHD	54	44	RIA (DiaSorin)	Median 60.7	Max 20	Age, season, region, sex, race, diabetes, on blood pressure medications, income, taking vitamin D supplements, physical activity, alcohol, BMI, blood pressure, CRP, eGFR, albumin:creatinine urinary secretion ratio, serum lipoprotein(a) (for all-cause mortality), serum thyroxine, total serum carotenoids (all-cause mortality), serum iron, RBC folate, serum vitamin C, serum vitamin A, serum vitamin E, serum homocysteine, serum total calcium, serum alpha-carotene (for CHD mortality), serum lycopene (for CHD mortality)
Donneyomg et al., 2016 [63]	NHANES III, U.S. 1988-1994 Follow-up to 2006	CVD	Range 30-90	46.5	RIA (DiaSorin)	NR	Mean 12.9	Age, ethnicity/race, sex, season of blood draw, census region of residence, BMI, hypertension, hyperlipidaemia, diabetes, vitamin D supplement use, smoking status, CRP levels, eGFR, stroke, MI, heart failure, cancer (except skin cancer), self-rated health status, outdoor recreational activity, and intensity of physical activity

First Author, Publication Year [Reference]	Study Name, Location	Mortality Outcomes	Mean Age (Years)	% Men	Vitamin D Assay	Mean 25(OH)D (nmol/L)	Follow-Up Time (years)	Adjustments
Schmutz et al., 2016 [64]	NHANES III, U.S. 1988-1994 follow-up to 2006	All-cause<br>CVD<br>Cancer<br>Non-cancer/non-CVD	43.4	48.8	RIA (DiaSorin)	Median 70.6	Median 14.5	Age, sex, race/ethnicity, season, poverty income ratio, BMI, physical activity, smoking status, alcohol consumption, education, hormone replacement therapy, hypertension, diabetes mellitus, hypercholesterolemia, history of MI, history of stroke, and history of cancer
Durazo-Arvizu et al., 2017 [65]	NHANES III, U.S. 1988-1994<br>Follow-up to 2006	All-cause	≥20	NR	RIA (DiaSorin), measurements standardised to LC-MS/MS reference measurement procedures	NR	NR	Age, sex, race/ethnicity, season
Ford et al., 2011 [66]	NHANES 2001-2004, U.S.	All-cause	45.9	51.3	RIA (DiaSorin)	60.6	Median 3.8	Age, sex, race or ethnicity, educational status, smoking status, alcohol intake, leisure-time physical activity, vitamin or mineral or supplement use, SBP, non-HDL cholesterol, HDL cholesterol, HbA1c, CRP, albuminuria, serum calcium, waist circumference, histories of CVD, cancer and diabetes, and 6-month examination period
Amer et al., 2013 [67]	NHANES 2001-2004, U.S.	All-cause<br>CVD	46.6	48.2	RIA (DiaSorin)	Median 52.4	Median 3.8	Race, age, sex, hypertension, smoking status, CRP, obesity, total cholesterol, renal function, and serum glucose
Formiga et al., 2014 [68]	Octabaix Study, Spain	All-cause<br>CVD	85	39.4	RIA (DiaSorin)	69.9	Median 2.8	Unadjusted
Buchebner et al., 2016 [69]	Malmö Osteoporosis Prospective Risk Assessment (OPRA) cohort, Sweden	All-cause	75.2	0	LC-MS/MS	62	Max 15	BMI, physical activity, smoking, osteoporosis, and comorbidities (CVD, respiratory disease, kidney disease, diabetes mellitus)<br>*For analyses from age 75, comorbidities were not adjusted for because data on comorbidities were not available*
Leu Agelii et al., 2017 [70]	Population Study of Women in Gothenburg, Sweden	All-cause	47.0<br>Range 38-60	0	ECLIA (Roche Diagnostics)	Median 69.0	Max 37	Season, waist circumference, leisure time physical activity, hypertension, triglyceride level, age, smoking, and occupational class
Masson et al., 2015 [71]	PREDICTOR, Italy	All-cause	72.9	52.6	ECLIA (Roche Diagnostics)	Median 30.7	Mean 3.8	Age, sex, season, eGFR, SBP, history of diabetes mellitus, COPD, alcohol consumption, atrial fibrillation, heart failure, smoking, dyslipidaemia, history of ischaemic heart disease (angina, previous MI or revascularisation procedures)

First Author, Publication Year [Reference]	Study Name, Location	Mortality Outcomes	Mean Age (Years)	% Men	Vitamin D Assay	Mean 25(OH)D (nmol/L)	Follow-Up Time (years)	Adjustments
Trevisan et al., 2017 [72]	Progetto Veneto Anziani (Pro.V.A.), Italy	All-cause	74.4	40.3	NR	NR	Mean 4.4	Sex, age, BMI, diabetes mellitus, anaemia, CVD, osteoarthritis, cancer, cognitive impairment, smoking, education level, income, vision, hearing, activity of daily living and instrumental activity of daily living scales, dependencies, Short Physical Performance Battery score, daily medications
Jassal et al., 2010 [73]	Rancho Bernardo Study, U.S.	CVD	74	38	CLIA	104.8	Mean 6.4<br>Median 6.8	Age, sex, BMI, SBP, LDL cholesterol, fasting glucose, exercise, log (urine albumin/creatinine ratio), prevalent CVD, season of blood draw, current use of diuretics, calcium channel blockers, beta-blockers, angiotensin-converting enzyme inhibitor
Dudenkov et al., 2018 [74]	Rochester Epidemiology Project database, U.S.	All-cause<br>CVD<br>Cancer<br>Respiratory<br>Non-cancer/non-CVD/non-respiratory	54.3	22.9	LC-MS/MS	74.9	Median 4.8	Age, sex, race/ethnicity, month, Charlson comorbidity index score
Signorello et al., 2013 [75]	Southern Community Cohort Study (SCCS), U.S.	All-cause<br>CVD<br>Cancer<br>Non-CVD/non-cancer/other non-external causes	Range 40-79	58.0	CLIA (LIAISON® 25 OH Vitamin D TOTAL assay, DiaSorin)	Men 42.9<br>Women 36.7	Mean 3.6	Sex, race, age, enrolment site, date of blood collection, BMI, smoking, physical activity, and household income
Samefors et al., 2014 [76]	Study of Health and Drugs in the Elderly (SHADES), Sweden<br>*Nursing home residents*	All-cause	Men 83<br>Women 86	32.1	HPLC with UV detection<br>*25(OH)D_3 only*	40.2	Mean 3	Age, sex, BMI, SBP, DBP, cystatin C eGFR, season of blood sample collection, time living in the nursing home, previous CVD, neoplastic diseases, dementia, and physical activity level
Tunstall-Pedoe et al., 2015 [77]	Scottish Heart Health Extended Cohort (SHHEC), Scotland	All-cause<br>CVD	Range 25-64	48.7	CLIA (ARCHITECT, Abbott Laboratories)	Median 36.4	Max 22	Season, age, sex, family history of CHD, Scottish Index of Multiple Deprivation, tobacco smoking dose, total cholesterol and HDL cholesterol
Domiciano et al., 2016 [78]	Sao Paulo Ageing & Health (SPAH) Study, Brazil	All-cause<br>CVD	73.3	38.4	RIA (DiaSorin)	74.9	Mean 4.1	Unadjusted<br>*25(OH)D increment for adjusted results not reported*
Hutchinson et al., 2010 [79]	Fourth Tromsø study, Norway	All-cause<br>CVD<br>Cancer	58.9	38.9	ECLIA (Roche Diagnostics)<br>*25(OH)D_3 only*	Non-smokers 52.3<br>Smokers 72.0	Mean 11.7	Age, sex, BMI, physical activity score, diabetes, hypertension, creatinine, prior CVD, and prior cancer

First Author, Publication Year [Reference]	Study Name, Location	Mortality Outcomes	Mean Age (Years)	% Men	Vitamin D Assay	Mean 25(OH)D (nmol/L)	Follow-Up Time (years)	Adjustments
Michaëlsson et al., 2010 [80]	Uppsala Longitudinal Study of Adult Men (ULSAM), Sweden	All-cause<br>CVD<br>Cancer<br>Gastrointestinal cancers<br>Enterohepatic cancers (liver, bile duct, pancreas, small intestine, colon, and rectum)<br>Pancreatic, liver, and biliary duct cancers<br>Non-cancer	71	100	HPLC MS	NR	Median 12.7	Age, weight, height, calcium intake, season of blood draw, social class, smoking status, leisure physical activity, self-perceived health, presence of diabetes mellitus, other endocrine disease, hematologic diseases, dermatoses, infectious disease, musculoskeletal disease, psychiatric disease, respiratory disease, kidney or urinary disease, gastrointestinal disease, supplemental vitamin D use, total vitamin D intake, fish intake, plasma PTH, plasma cystatin C, plasma CRP, serum calcium, serum phosphate, plasma troponin I, plasma N-terminal pro brain natriuretic peptide, plasma cholesterol, plasma triglycerides, plasma HDL cholesterol, plasma retinol, plasma insulin, total energy intake, alcohol intake, SBP, DBP, lipid-lowering treatment, and antihypertensive treatment
Tomson et al., 2013 [81]	Whitehall study, UK	All-cause<br>CVD<br>IHD<br>Stroke<br>Cancer<br>Respiratory diseases<br>Non-CVD<br>Non-cancer/non-CVD/non-respiratory	76.9	100	IDS-iSYS immunoassay (Immunodiagnostic Systems)	Median 56	Mean 13.1	Month of blood collection, age, recall of a diagnosis of IHD, stroke, cancer, or diabetes, self-reported health/frailty, smoking status, drinking status, employment grade, LDL-cholesterol, HDL-cholesterol, apolipoprotein A_1, apolipoprotein B, BMI, blood pressure, albumin, fibrinogen, CRP, and eGFR
Semba et al., 2009 [82]	Women's Health and Aging Study (WHAS) I and II, U.S.	All-cause	74	0	Radioreceptor assay (Nichols Institute Diagnostics)	Median 50.9	Median 6	Age, race, education, season, BMI, smoking, supplement use, physical activity, total cholesterol, HDL cholesterol, and chronic diseases (hypertension, diabetes, heart failure, stroke, and renal insufficiency)
Eaton et al., 2011 [83]	Women's Health Initiative (WHI), U.S.	All-cause<br>CVD<br>Cancer	66<br>Range 50-79	0	CLIA (DiaSorin)	Median 50.0	10	Month, age, ethnicity, Calcium and vitamin D trial indicator, smoking status, fracture at ≥55 y of age, waist circumference, BMI, physical activity, and (for all-cause and CVD mortality) weekly alcohol consumption, history of cancer, hypertension, treated diabetes, CVD, SBP, and (for cancer mortality) education, current aspirin use, and use of vitamin D supplements

First Author, Publication Year [Reference]	Study Name, Location	Mortality Outcomes	Mean Age (Years)	% Men	Vitamin D Assay	Mean 25(OH)D (nmol/L)	Follow-Up Time (years)	Adjustments
Kitamura et al., 2010 [84]	Yamato Study, Japan *Elderly people requiring home care*	All-cause	83.6	30.8	CLIA , (Nichols Advantage®, Nichols Institute Diagnostics)	Median 47.9	2	Unadjusted

[1] Eight European centres: Florence, Italy; Leuven, Belgium; Malmö, Sweden; Manchester, UK; Santiago de Compostela, Spain; Łódź, Poland; Szeged, Hungary; Tartu, Estonia.

BMD, bone mineral density; BMI, body mass index; CHD, coronary heart disease; CLIA, competitive chemiluminescent immunoassay; CRP, C-reactive protein; CVD, cardiovascular disease; DBP, diastolic blood pressure; ECLIA, Enhanced chemiluminescence immunoassay; EPIC, European Prospective Investigation into Cancer and Nutrition; ESTHER, Epidemiologische Studie zu Chancen der Verhütung, Früherkennung und optimierten Therapie chronischer Erkrankungen in der älteren Bevölkerung; FEV1, forced expiratory volume in 1 second; GFR, glomerular filtration rate; HDL, high-density lipoprotein; HPLC, high-performance liquid chromatography; HUNT, Helseundersokelsen i Nord-Trøndelag; IHD, ischaemic heart disease; InCHIANTI, Invecchiare in Chianti (Aging in the Chianti Area); KORA-Age, Cooperative Health Research in the Region of Augsburg-Age study (follow-up study of four Multinational Monitoring of Trends and Determinants in Cardiovascular disease (MONICA)/KORA surveys); LC-MS/MS, liquid chromatography-tandem mass spectrometry; LDL, low-density lipoprotein; MI, myocardial infarction; MMSE, Mini-Mental State Examination; MONICA, Multinational Monitoring of Trends and Determinants in Cardiovascular Disease; MS, mass spectrometry; N/A, not applicable/no study name; NHANES, National Health and Nutrition Examination Survey; NHANES III, Third National Health and Nutrition Examination Survey; NR, not reported; PASE, Physical Activity Scale for the Elderly; PPT, Reuben's Physical Performance Test; PREDICTOR, Valutazione della PREvalenza di DIsfunzione Cardiaca asinTOmatica e di scompenso caRdiaco conclamato in un campione di popolazione di età ≥ 65 anni nel Lazio; PTH, parathyroid hormone; RIA, radioimmunoassay; SBP, systolic blood pressure

Table S2: Results from prospective studies of circulating 25-hydroxyvitamin D and all-cause mortality.

Study Name, Location	Author, Publication Year	Number of Participants	Number of Deaths	Estimate	25(OH)D Contrast/Metric	Estimate (95% CI)
N/A, New Zealand	Bolland et al., 2010	1471	63	HR	<50 versus ≥50 nmol/L	0.9 (0.5-1.6)
N/A, Nursing home residents, Spain	Centeno Peláez et al., 2014	74	8	OR	<12.48 versus ≥12.48 nmol/L	19.7 (1.48-261.53)
N/A, Scotland	Jia et al., 2007	398	129	HR	Lowest quintile (men 6.0-23.0; women 7.0-19.0 nmol/L) versus highest quintile (men 47.1-82.0; women 39.1-82.0 nmol/L)	1.74 (0.91-3.34)
N/A, Japan	Kuroda et al., 2009	1232	107	HR	<50 versus ≥50 nmol/L	2.17 (1.27-3.72)
N/A, Nursing home residents, Austria	Pilz et al., 2012	961	284	HR	Lowest quartile (<14.0 nmol/L) versus highest quartile (>25.5 nmol/L)	1.56 (1.01-2.40)
Busselton Health Survey, Australia	Zhu et al., 2018	3946	889	HR	Per SD (18 nmol/L) increment<br><50 versus 50-75 nmol/L<br>>75 versus 50-75 nmol/L	0.84 (0.77-0.91)<br>1.29 (1.11-1.50)<br>1.05 (0.85-1.29)
Chin-Shan Community Cardiovascular Cohort Study (CCCC), Taiwan	Chien et al., 2015	1816	559	HR	Highest quartile (≥63.8 nmol/L) versus lowest quartile (<39.0 nmol/L)	0.90 (0.67-1.21)
Copenhagen City Heart Study (CCHS), Denmark	Brøndum-Jacobsen et al., 2012	10,170	6747	HR	<25 versus ≥75 nmol/L	1.37 (1.23-1.54)
Copenhagen City Heart Study (CCHS), Denmark	Afzal et al., 2014	9902	7132	HR	Per 20 nmol/L lower	1.17 (1.12-1.24)
Copenhagen General Population Study (CGPS), Denmark	Afzal et al., 2014	25,432	1386	HR	Per 20 nmol/L lower	1.20 (1.06-1.37)
Concord Health and Ageing in Men Project (CHAMP), Australia	Hirani et al., 2014	1659	355	HR	<50 versus ≥75 nmol/L	1.40 (1.04-1.89)
Cardiovascular Health Study (CHS), U.S.	Kestenbaum et al., 2011	2312	1226	HR	<37.4 versus >74.9 nmol/L<br>Per 25 nmol/L lower	1.29 (1.05-1.57)<br>1.09 (1.02-1.17)
Cardiovascular Health Study (CHS), U.S.	de Boer et al., 2012	1621	826	HR	Low (<lowest season-specific 29th percentile; <43, 50, 61, and 55 nmol/L in winter, spring, summer, and autumn, respectively) versus normal	1.32 (1.14-1.53)
Clalit Health Services (CHS) cohort, Israel	Saliba et al., 2012	182,152	7247	HR	Lowest quartile (≤33.8 nmol/L) versus highest quartile (>65.2 nmol/L)<br><30 versus ≥75 nmol/L	1.85 (1.70-2.01)<br>1.81 (1.64-2.01)
Clalit Health Services (CHS) cohort, Israel	Saliba et al., 2014	175,781	12,337	HR	Per 10 nmol/L increase	0.91 (0.90-0.92)
Copenhagen vitamin D (CopD) study, Denmark	Durup et al., 2012	247,574	15,198	HR	10 versus 50 nmol/L<br>140 versus 50 nmol/L	2.13 (2.02-2.24)<br>1.42 (1.31-1.53)
Danish Osteoporosis prevention Study (DOPS), Denmark	Schierbeck et al., 2012	2013	135	HR	<50 versus ≥50 nmol/L	1.18 (0.84-1.67)
European Male Ageing Study (EMAS), Europe	Lee et al., 2014	2816	187	HR	Lowest quartile (<40.0 nmol/L) versus highest quartile (>79.1 nmol/L)<br><25 nmol/L versus ≥75 nmol/L<br><30 nmol/L versus >50 nmol/L<br>Per SD decrease	2.37 (1.33-4.24)<br>2.28 (1.20-4.34)<br>1.98 (1.25-3.12)<br>1.45 (1.16-1.81)
EPIC-Norfolk, UK	Khaw et al., 2014	14,641	2776	HR	≥90 versus <30 nmol/L<br>Per 20 nmol/L increase	0.73 (0.59-0.90)<br>0.92 (0.88-0.96)

Study Name, Location	Author, Publication Year	Number of Participants	Number of Deaths	Estimate	25(OH)D Contrast/Metric	Estimate (95% CI)
ESTHER, Germany	Schöttker et al., 2013	9578	1083	HR	<30 versus >50 nmol/L	1.71 (1.43-2.03)
ESTHER, Germany	Schöttker et al., 2014	9579	1450	HR	<30 versus ≥50 nmol/L	
					Not time-dependent	1.54 (1.32-1.80)
					Time-dependent [1]	1.60 (1.37-1.86)
ESTHER, Germany	Brenner et al., 2017	9579	1646	HR	Per 20 nmol/L increase	0.90 (0.86-0.95)
General Population Trial of Linxian, China	Lin et al., 2012	1101	793	HR	Highest quartile (≥48.4 nmol/L) versus lowest quartile (<19.6 nmol/L)	1.07 (0.88-1.30)
					Per 15 nmol/L increase	1.01 (0.97-1.05)
Health ABC, U.S.	Kritchevsky et al., 2012	2638	691	HR	<25 versus ≥75 nmol/L	2.27 (1.59-3.24)
		1615	373		Whites	2.02 (1.02-3.99)
		1023	318		Blacks	2.59 (1.57-4.26)
Health in Men Study (HIMS), Australia	Wong et al., 2013	4203	1144	HR	Lowest quartile (10.0-52.8 nmol/L) versus highest quartile (81.7-238.4 nmol/L)	1.20 (1.02-1.42)
					Per 10 nmol/L decrease	1.04 (1.01-1.07)
Hoorn Study, the Netherlands	Pilz et al., 2009	614	51	HR	Lowest (mean 30.6 nmol/L) versus upper three sex-specific quartiles	1.97 (1.08-3.58)
Nord-Trøndelag Health Study 2 (HUNT2), Norway	Sun et al., 2017	6377	1539	HR	Lowest quartile (<34.5 nmol/L) versus highest quartile (≥58.1 nmol/L)	1.27 (1.09-1.48)
					<25 versus 50-74.9 nmol/L	1.41 (1.14-1.74)
					≥75 versus 50-74.9 nmol/L	1.08 (0.86-1.34)
InCHIANTI, Italy	Semba et al., 2010	1006	228	HR	Lowest quartile (<26.2 nmol/L) versus highest quartile (>63.9 nmol/L)	2.11 (1.22-3.64)
Kuopio Ischaemic Heart Disease Risk Factor (KIHD) Study, Finland	Virtanen et al., 2011	1136	87	HR	Lowest tertile (8.9-34.0 nmol/L) versus highest tertile (50.9-112.8 nmol/L)	2.06 (1.12-3.80)
KORA-Age study, Germany	Vogt et al., 2015	954	98	OR	<37.4 versus ≥74.9 nmol/L	3.39 (1.08-10.65)
Longitudinal Aging Study Amsterdam (LASA), the Netherlands	El Hilali et al., 2016	1317	986	HR	<25 versus ≥75 nmol/L	1.46 (1.12-1.91)
Longitudinal Aging Study Amsterdam (LASA), the Netherlands	Visser et al., 2006	1260	380	HR	<25 versus ≥75 nmol/L	1.28 (0.85-1.92)
					Per SD (23.9 nmol/L) increase	0.91 (0.81-1.02)
Melbourne Collaborative Cohort Study (MCCS), Australia	Heath et al., 2017	4964	2307	HR	Highest quintile (median 77.0 nmol/L) versus lowest quintile (median 30.3 nmol/L)	0.67 (0.54-0.84)
					Per 25 nmol/L increment	0.86 (0.78-0.96)
MIDSPAN Family Study, Scotland	Welsh et al., 2012	1492	70	HR	<37.5 nmol/L versus ≥37.5 nmol/L	2.02 (1.17-3.51)
					Per SD increase	0.74 (0.56-0.99)
MINOS study, France	Szulc et al., 2009	782	182	HR	Lowest quartile (<65 nmol/L summer, <40 nmol/L other seasons)	1.70 (1.01-2.84)
					versus highest quartile (>97.5 nmol/L summer, >77.5 nmol/L other seasons)	1.44 (1.03-2.03)
					Lowest quartile (<65 nmol/L summer, <40 nmol/L other seasons) versus upper three quartiles (≥65 nmol/L summer, ≥40 nmol/L other seasons)	1.22 (1.01-1.48)
					Per SD decrease	
MONICA, Switzerland	Rohrmann et al., 2013	3191	459	HR	Highest quartile (62.2-249.1 nmol/L) versus lowest quartile (0-33.5 nmol/L)	0.67 (0.52-0.87)
					≥100 versus <25 nmol/L	0.46 (0.24-0.88)
					Per 25 nmol/L increase	0.83 (0.74-0.92)

Study Name, Location	Author, Publication Year	Number of Participants	Number of Deaths	Estimate	25(OH)D Contrast/Metric	Estimate (95% CI)
Monica10 study, Denmark	Skaaby et al., 2013a	2441	635	HR	Highest versus lowest quartile	0.73 (0.57-0.93)
					Per 10 nmol/L increase	0.96 (0.93-0.99)
Monica10 and Inter99 (combined), Denmark	Skaaby et al., 2013b	8329	633	HR	Highest versus lowest quartile	0.73 (0.57-0.92)
					Per 10 nmol/L increase	0.95 (0.92-0.99)
Osteoporotic Fractures in Men Study (MrOS), Sweden	Johansson et al., 2012	2878	577	HR	Per SD decrease in *z*-score (gradient of risk)	1.05 (0.96-1.14)
Osteoporotic Fractures in Men (MrOS) study, U.S.	Cawthon et al., 2010	1490	330	HR	Lowest quartile (<49.7 nmol/L) versus highest quartile (≥74.9 nmol/L)	0.95 (0.68-1.34)
					Per SD (19.9 nmol/L) decrease	1.01 (0.89-1.14)
National Diet and Nutrition Survey (NDNS), UK	Bates et al., 2012	1054	717	HR	Per SD (men 27.7 nmol/L, women 23.7 nmol/L) increase	0.89 (0.82-0.98)
Newcastle 85+ Study, UK	Granic et al., 2015	775	363	HR	Season-specific quartiles:	
					Lowest versus middle two quartiles	1.10 (0.85-1.42)
		304	169		Men	0.95 (0.65-1.39)
		471	194		Women	1.27 (0.87-1.84)
					Highest versus middle two quartiles	1.25 (0.97-1.63)
					Men	1.06 (0.69-1.64)
					Women	1.51 (1.06-2.14)
NHANES III, U.S.	Melamed et al., 2008	13,331	1806	Rate ratio	Lowest quartile (<44.4 nmol/L) versus highest quartile (>80.1 nmol/L)	1.26 (1.08-1.46)
NHANES III, U.S.	Ginde et al., 2009	3265	1493	HR	<25 versus ≥100 nmol/L	1.83 (1.14-2.94)
					Per 10 nmol/L	0.95 (0.92-0.98)
NHANES III, U.S.	Smit et al., 2012	4731	NR	HR	Lowest quartile (<49.5 nmol/L) versus highest quartile (>84.1 nmol/L)	1.27 (1.09-1.47)
NHANES III, U.S.	Deng et al., 2013	16,819	3703	HR	≥100 versus <50 nmol/L	0.79 (0.66-0.93)
NHANES III, U.S.	Sempos et al., 2013	11,315	3784	RR	<20 versus 75-99 nmol/L	1.6 (1.2-2.2)
					≥120 versus 75-99 nmol/L	1.4 (0.9-2.2)
NHANES III, U.S.	Ford et al., 2015	10,795	1792	HR	<25 versus ≥75 nmol/L	1.37 (0.91-2.05)
					<30 versus ≥50 nmol/L	1.47 (1.06-2.04)
NHANES III, U.S.	Daraghmeh et al., 2016	10,517	NR	HR	Highest quartile (80.1-400.1 nmol/L) versus lowest quartile (8.7-43.9 nmol/L)	0.74 (0.57-0.96)
NHANES III, U.S.	Schmutz et al., 2016	15,998	3890	HR	≥100 versus <40 nmol/L	0.70 (0.50-0.83)
					Highest quartile (>79.9 nmol/L) versus lowest quartile (<44.2 nmol/L)	0.77 (0.68-0.87)
					Per 25 nmol/L increase	0.93 (0.89-0.97)
NHANES III, U.S.	Durazo-Arvizu et al., 2017	15,099	3784	RR	<20 versus 75-99 nmol/L	2.1 (1.6-2.7)
					110-119 versus 75-99 nmol/L	1.1 (0.6-2.1)
NHANES 2001-2004, U.S.	Ford et al., 2011	7531	347	HR	<50 versus ≥75 nmol/L	1.28 (0.86-1.90)
					Per 10 nmol/L increase	0.93 (0.86-1.01)
NHANES 2001-2004, U.S.	Amer et al., 2013	10,170	513	HR	Per 25 nmol/L increase:	
					for 25(OH)D ≤52.4 nmol/L	0.54 (0.35-0.84)
					for 25(OH)D >52.4 nmol/L	0.83 (0.63-1.11)
Octabaix study, Spain	Formiga et al., 2014	312	58	HR [2]	Lowest quartile (<34.9 nmol/L) versus highest quartile (>83.4 nmol/L)	1.28 (0.61-2.6)

Study Name, Location	Author, Publication Year	Number of Participants	Number of Deaths	Estimate	25(OH)D Contrast/Metric	Estimate (95% CI)
Malmö Osteoporosis Prospective Risk Assessment (OPRA) cohort, Sweden	Buchebner et al., 2016	1011 at age 75	565 from age 75	HR	Low (<50 nmol/L) versus high (>75 nmol/L)	
					10-year risk from age 75	1.4 (1.0-1.9)
					5-year risk from age 75	1.4 (0.8-2.5)
		642 at age 80	307 from age 80		10-year risk from age 80	1.9 (1.4-2.6)
					5-year risk from age 80	2.1 (1.2-3.6)
		348 at age 85	105 from age 85		5-year risk from age 85	2.0 (1.1-3.7)
Population Study of Women in Gothenburg, Sweden	Leu Agelii et al., 2017	1227	635	HR	Lowest quartile (≤51.45 nmol/L) versus upper three quartiles (>51.45 nmol/L)	
					37 years of follow-up (max duration)	1.42 (1.17-1.72)
			456		32 years of follow-up	1.43 (1.14-1.80)
			312		27 years of follow-up	1.39 (1.06-1.83)
			202		22 years of follow-up	1.44 (1.02-2.03)
			114		17 years of follow-up	1.96 (1.25-3.08)
PREDICTOR, Italy	Masson et al., 2015	1835	135	HR	Per SD increase in log 25(OH)D	0.70 (0.53-0.94)
Progetto Veneto Anziani (Pro.V.A.), Italu	Trevisan et al., 2017	2925	745	OR	<75 versus ≥75 nmol/L	1.42 (1.31-1.53)
Rochester Epidemiology Project, U.S.	Dudenkov et al., 2018	11,022	723	HR	<30 versus whites with 50-125 nmol/L	
		9653	648		White race/ethnicity	2.52 (2.17-2.91)
		1369	75		Other race/ethnicity	1.69 (1.10-2.62)
					>125 versus whites with 50-125 nmol/L	
					White race/ethnicity	1.04 (0.81-1.33)
					Other race/ethnicity	2.06 (0.77-5.51)
Southern Community Cohort Study (SCCS), U.S.	Signorello et al., 2013	3704	1852	OR	Lowest quartile (<25.4 nmol/L) versus highest quartile (>54.0 nmol/L)	1.80 (1.43-2.27)
		2550	1275		African Americans	1.60 (1.20-2.14)
		1132	566		Non-African Americans	2.11 (1.39-3.21)
Study of Health and Drugs in the Elderly (SHADES), Sweden *Nursing home residents*	Samefors et al., 2014	333	147	HR	Lowest quartile (11-29 nmol/L) versus highest quartile (48-120 nmol/L)	2.02 (1.31-3.12)
Scottish Heart Health Extended Cohort (SHHEC), Scotland	Tunstall-Pedoe et al., 2015	11,628	2928	HR	Lowest quintile (<27.1 nmol/L) versus highest quintile (≥53.8 nmol/L)	1.22 (1.08-1.39)
		5658	1715		Men	1.39 (1.18-1.64)
		5970	1213		Women	1.13 (0.93-1.37)
					Per 20 nmol/L decrease	1.10 (1.05-1.15)
					Men	1.12 (1.06-1.18)
					Women	1.07 (0.99-1.15)
Sao Paulo Ageing & Health (SPAH) Study, Brazil	Domiciano et al., 2016	832	131	HR [2]	Lowest quartile (<32.4 nmol/L) versus highest quartile (≥62.4 nmol/L)	1.94 (1.22-3.10) [a]
					<50 versus ≥75 nmol/L	1.09 (0.64-1.83) [a]
Fourth Tromsø study, Norway	Hutchinson et al., 2010	7161	1359	HR	Lowest (mean non-smokers 33.8, smokers 49.2 nmol/L) versus highest quartile (mean non-smokers 72.3, smokers 97.5 nmol/L):	
		4751	798		Non-smokers [3]	1.32 (1.07-1.62)
		2410	561		Smokers [3]	1.06 (0.83-1.35)
Uppsala Longitudinal Study of Adult Men (ULSAM), Sweden	Michaëlsson et al., 2010	1194	584	HR	<5th percentile (<39 nmol/L) versus 10th–90th percentile (46-93 nmol/L)	1.33 (0.93-1.88)
					<10th percentile (<46 nmol/L) versus 10th–90th percentile (46-93 nmol/L)	1.43 (1.11-1.84)
					>90th percentile (>93 nmol/L) versus 10th–90th percentile (46-93 nmol/L)	1.27 (0.97-1.66)
					>95th percentile (>98 nmol/L) versus 10th–90th percentile (46-93 nmol/L)	1.67 (1.19-2.35)

Study Name, Location	Author, Publication Year	Number of Participants	Number of Deaths	Estimate	25(OH)D Contrast/Metric	Estimate (95% CI)
Women's Health and Aging Study (WHAS) I and II, U.S.	Semba et al., 2009	714	100	HR	Lowest quartile (<38.2 nmol/L) versus highest quartile (>67.4 nmol/L)	2.45 (1.12-5.36)
Women's Health Initiative (WHI), U.S.	Eaton et al., 2011	2429	224	HR	Lowest quartile (3.3-36.5 nmol/L) versus highest quartile (65.4-146.7 nmol/L)	1.25 (0.80-1.95)
Whitehall study, UK	Tomson et al., 2013	5409	3215	RR	Two-fold higher concentration	0.78 (0.72-0.85)
Yamato Study, Japan	Kitamura et al., 2010	198	40	RR [2]	Lowest quartile (<36.9 nmol/L) versus highest quartile (≥64.4 nmol/L)	1.12 (0.52-2.40)

[1] Time-dependent analyses used repeated measurements of 25(OH)D and repeated measurements of covariates. [2] Unadjusted. [3] Results were reported separately for smokers and non-smokers because the immunoassay used in the Tromsø study appeared to overestimate 25(OH)D among smokers. HR, hazard ratio; N/A, not applicable/no study name; OR, odds ratio; RR, relative risk

Table S3: Results from prospective studies of circulating 25-hydroxyvitamin D and cardiovascular mortality.

Cause of Death<br>Study Name, Location	Author,<br>Publication Year	Number of Participants	Number of Deaths	Estimate	25(OH)D Contrast/Metric	Estimate (95% CI)
**Cardiovascular**						
Busselton Health Survey, Australia	Zhu et al., 2018	3946	363	HR	<50 versus 50-75 nmol/L	1.46 (1.16-1.85)
					>75 versus 50-75 nmol/L	1.20 (0.86-1.69)
					Per SD (18 nmol/L) increment	0.82 (0.72-0.94)
Copenhagen City Heart Study (CCHS), Denmark	Afzal et al., 2014	9902	2877	HR	Per 20 nmol/L lower	1.17 (1.08-1.28)
Copenhagen General Population Study (CGPS), Denmark	Afzal et al., 2014	25,432	317	HR	Per 20 nmol/L lower	1.32 (0.99-1.76)
Cardiovascular Health Study (CHS), U.S.	Kestenbaum et al., 2011	2312	389	HR	<37.4 versus >74.9 nmol/L	1.17 (0.83-1.67)
					Per 25 nmol/L lower	1.06 (0.94-1.19)
Copenhagen vitamin D (CopD) study, Denmark	Durup et al., 2015	243,672	5454	HR	~12.5 versus 70 nmol/L:	2.0 (1.8-2.1)
					Men	2.5 (2.2-2.9)
					Women	1.7 (1.5-1.9)
					~125 versus 70 nmol/L	1.3 (1.2-1.4)
European Male Ageing Study (EMAS),	Lee et al., 2014	2452	72	HR	Lowest quartile (<40 nmol/L) versus highest quartile (>79.1 nmol/L)	2.21 (0.75-6.51)
					<25 nmol/L versus ≥75 nmol/L	1.26 (0.40-3.70)
					Per SD decrease	1.38 (0.95-1.99)
EPIC-Norfolk, UK	Khaw et al., 2014	14,641	854	HR	≥90 versus <30 nmol/L	0.73 (0.49-1.09)
					Per 20 nmol/L increase	0.92 (0.85-0.99)
ESTHER, Germany	Perna et al., 2013	7709	176	HR	<30 versus ≥50 nmol/L	1.62 (1.07-2.48)
					Per 25 nmol/L increase	0.78 (0.65-0.93)
ESTHER, Germany	Schöttker et al., 2013	9554	350	HR	<30 versus >50 nmol/L	1.39 (1.02-1.89)
ESTHER, Germany	Schöttker et al., 2014	9506	407	HR	<30 nmol/L versus ≥50 nmol/L	
					Not time-dependent	1.20 (0.90-1.61)
					Time-dependent	1.41 (1.04-1.89)
ESTHER, Germany	Brenner et al., 2017	9554	552	HR	Per 20 nmol/L increase	0.89 (0.81-0.96)
General Population Trial of Linxian, China	Lin et al., 2012	1101	200	HR	Highest quartile (≥48.4 nmol/L) versus lowest quartile (<19.6 nmol/L)	0.93 (0.62-1.39)
					Per 15 nmol/L increase	0.98 (0.91-1.06)
Health ABC, U.S.	Kritchevsky et al., 2012	2638	228	HR	<25 versus ≥75 nmol/L	1.90 (0.97-3.72)
		1615	124		Whites	1.38 (0.40-4.80)
		1023	104		Blacks	2.47 (0.96-6.36)
Hoorn Study, the Netherlands	Pilz et al., 2009	614	20	HR	Lowest (mean 30.6 nmol/L) versus upper three sex-specific quartiles	5.38 (2.02-14.34)
InCHIANTI, Italy	Semba et al., 2010	1006	107	HR	Lowest quartile (<26.2 nmol/L) versus highest quartile (>63.9 nmol/L)	2.64 (1.14-4.79)
Kuopio Ischaemic Heart Disease Risk Factor (KIHD) Study, Finland	Virtanen et al., 2011	1136	35	HR	Lowest tertile (8.9-34.0 nmol/L) versus highest tertile (50.9-112.8 nmol/L)	1.79 (0.75-4.28)
Longitudinal Aging Study Amsterdam (LASA), the Netherlands	Eli Hilali et al., 2016	1317	236	HR	<25 versus ≥75 nmol/L	1.42 (0.80-2.51)
MIDSPAN Family Study, Scotland	Welsh et al., 2012	1492	12	HR	<37.5 nmol/L versus ≥37.5 nmol/L	8.13 (1.26-52.7)

Cause of Death<br>Study Name, Location	Author,<br>Publication Year	Number of Participants	Number of Deaths	Estimate	25(OH)D Contrast/Metric	Estimate (95% CI)
Mini-Finland Health Survey, Finland	Kilkkinen et al., 2009	6219	933	HR	Highest quintile (men 62.0-180.0, women 56.0-151.0 nmol/L) versus lowest quintile (men 5.0-28.0, women 4.0-25.0 nmol/L)	0.76 (0.61-0.95)
MONICA, Switzerland	Rohrmann et al., 2013	3191	122	HR	Highest quartile (62.2-249.1 nmol/L) versus lowest quartile (0-33.5 nmol/L)	0.60 (0.35-1.04)
					Women	0.42 (0.17-1.00)
					Men	1.00 (0.50-1.99)
					Per 25 nmol/L increase	0.89 (0.73-1.08)
					Women	0.68 (0.46-1.00)
					Men	0.97 (0.77-1.23)
Monica10 and Inter99 (combined), Denmark	Skaaby et al., 2012	8329	178	HR	Highest quartile (Monica10, 81-204; Inter99, 65-255 nmol/L) versus lowest quartile (Monica10, 13-45; Inter99, 10-32 nmol/L)	1.1 (0.70-1.7)
Osteoporotic Fractures in Men (MrOS) study, U.S.	Cawthon et al., 2010	1473	110	HR	Lowest quartile (<49.7 nmol/L) versus highest quartile (≥74.9 nmol/L)	1.52 (0.83-2.80)
					Per SD (19.9 nmol/L) decrease	1.24 (0.99-1.55)
NHANES III, U.S.	Melamed et al., 2008	13,331	777	Rate ratio	Lowest quartile (<44.4 nmol/L) versus highest quartile (>80.1 nmol/L)	1.20 (0.87-1.64)
NHANES III, U.S.	Ginde et al., 2009	3265	767	HR	<25 versus ≥100 nmol/L	2.36 (1.17-4.75)
NHANES III, U.S.	Fiscella et al., 2010	15,363	933	Incident rate ratio	Highest quartile (>80 nmol/L) versus lowest quartile (<45 nmol/L)	0.79 (0.62-1.01)
NHANES III, U.S.	Liu et al., 2012	13,131	1451	HR	<50 versus ≥75 nmol/L	1.52 (1.29-1.79)
NHANES III, U.S.	Deng et al., 2013	16,819	1615	HR	≥100 versus <50 nmol/L	0.73 (0.56-0.97)
NHANES III, U.S.	Sempos et al., 2013	11,315	1660	RR	<20 versus 75-99 nmol/L	2.0 (1.2-3.5)
					≥120 versus 75-99 nmol/L	0.6 (0.2-1.7)
NHANES III, U.S.	Donneyong et al., 2016	11,746	1519	HR	≥75 versus <50 nmol/L	0.75 (0.62-0.91)
					Participants without CVD at baseline	0.67 (0.54-0.84)
NHANES III, U.S.	Schmutz et al., 2016	15,998	1715	HR	Highest quartile (>79.9 nmol/L) versus lowest quartile (<44.2 nmol/L)	0.79 (0.67-0.94)
					Per 25 nmol/L increase	0.92 (0.86-0.98)
NHANES 2001-2004, U.S.	Amer et al., 2013	10,170	186	HR	Per 25 nmol/L increase:	
					for 25(OH)D ≤52.4 nmol/L	0.50 (0.26-0.98)
					for 25(OH)D >52.4 nmol/L	0.83 (0.47-1.47)
Octabaix, Spain	Formiga et al., 2014	312	25	HR [1]	Lowest quartile (<34.9 nmol/L) versus highest quartile (>83.4 nmol/L)	1.04 (0.33-3.24)
Rancho Bernardo Study, U.S.	Jassal et al., 2010	1073	111	HR	Per SD (34.9 nmol/L) increase	1.07 (0.86-1.33)
Rochester Epidemiology Project, U.S.	Dudenkov et al., 2018	11,022	125	HR	<30 versus 50-125 bnol/L	2.48 (1.53-4.02)
					>125 versus 50-125 bnol/L	0.68 (0.25-1.87)
Southern Community Cohort Study (SCCS), U.S.	Signorello et al., 2013			OR	Lowest quartile (<25.4 nmol/L) versus highest quartile (>54.0 nmol/L)	
		754	377		African Americans	2.53 (1.44-4.46)
		308	154		Non-African Americans	3.25 (1.33-7.93)

Cause of Death<br>Study Name, Location	Author, Publication Year	Number of Participants	Number of Deaths	Estimate	25(OH)D Contrast/Metric	Estimate (95% CI)
Scottish Heart Health Extended Cohort (SHHEC), Scotland	Tunstall-Pedoe et al., 2015	11,628	1368	HR	Lowest quintile (<27.1 nmol/L) versus highest quintile (≥53.8 nmol/L)	1.48 (1.21-1.79)
		5658	823		Men	1.80 (1.40-2.33)
		5970	545		Women	1.25 (0.92-1.69)
					Per 20 nmol/L decrease	1.17 (1.09-1.25)
					Men	1.21 (1.11-1.32)
					Women	1.09 (0.97-1.23)
Sao Paulo Ageing & Health (SPAH) Study, Brazil	Domiciano et al., 2016	832	57	HR [1]	Lowest quartile (<32.4 nmol/L) versus highest quartile (≥62.4 nmol/L)	1.65 (0.84-3.22) [a]
					<50 versus ≥75 nmol/L	1.27 (0.54-3.04) [a]
Fourth Tromsø study, Norway	Hutchinson et al., 2010	7161	513	HR	Lowest (mean non-smokers 33.8, smokers 49.2 nmol/L) versus highest quartile (mean non-smokers 72.3, smokers 97.5 nmol/L):	
		4751	325		Non-smokers[b]	1.08 (0.79-1.48)
		2410	188		Smokers[b]	0.93 (0.61-1.44)
Uppsala Longitudinal Study of Adult Men (ULSAM), Sweden	Michaëlsson et al., 2010	1194	177	HR	<5th percentile (<39 nmol/L) versus 10th–90th percentile (46-93 nmol/L)	1.11 (0.54-2.26)
					<10th percentile (<46 nmol/L) versus 10th–90th percentile (46-93 nmol/L)	1.53 (0.97-2.41)
					>90th percentile (>93 nmol/L) versus 10th–90th percentile (46-93 nmol/L)	1.16 (0.69-1.93)
					>95th percentile (>98 nmol/L) versus 10th–90th percentile (46-93 nmol/L)	1.41 (0.72-2.78)
Whitehall study, UK	Tomson et al., 2013	5409	1358	RR	Two-fold higher concentration	0.80 (0.70-0.91)
Women's Health Initiative (WHI), U.S.	Eaton et al., 2011	2429	79	HR	Lowest (3.3-36.5 nmol/L) versus highest quartile (65.4-146.7 nmol/L)	1.27 (0.81-1.99)
**Coronary heart disease/Ischaemic heart disease/myocardial infarction**						
Copenhagen City Heart Study (CCHS), Denmark	Brøndum-Jacobsen et al., 2012	10,170	1522	HR	<25 versus ≥75 nmol/L	1.53 (1.18-1.98)
Copenhagen City Heart Study (CCHS) and Copenhagen General Population Study (CGPS) (combined), Denmark	Afzal et al., 2014	35,334	1424	HR	Per 20 nmol/L lower	1.21 (1.07-1.38)
Copenhagen vitamin D (CopD) study, Denmark	Durup et al., 2015	243,672	702	HR	~12.5 versus 70 nmol/L	2.1 (1.7-2.7)
					~125 versus 70 nmol/L	1.6 (1.2-2.0)
ESTHER, Germany	Perna et al., 2013	7709	79	HR	<30 versus ≥50 nmol/L	1.60 (0.84-3.06)
					Per 25 nmol/L increase	0.70 (0.53-0.92)
Mini-Finland Health Survey, Finland	Kilkkinen et al., 2009	6219	640	HR	Highest quintile (men 62.0-180.0, women 56.0-151.0 nmol/L) versus lowest quintile (men 5.0-28.0, women 4.0-25.0 nmol/L)	0.91 (0.70-1.18)
NHANES III, U.S.	Daraghmeh et al., 2016	10,517	NR	HR	Highest (80.1-400.1 nmol/L) versus lowest quartile (8.7-43.9 nmol/L)	0.59 (0.37-0.94)
Whitehall study, UK	Tomson et al., 2013	5409	659	RR	Two-fold higher concentration	0.84 (0.70-1.02)
**Sudden cardiac death**						
Cardiovascular Health Study (CHS), U.S.	Deo et al., 2011	2283	73	HR	<50 versus ≥50 nmol/L	1.47 (0.88-2.46)
					Per 12.5 nmol/L lower	1.12 (0.98-1.29)
**Heart failure**						
NHANES III, U.S.	Liu et al., 2012	13,131	101	HR	<50 versus ≥75 nmol/L	2.06 (1.01-4.25)
**Cerebrovascular disease/stroke**						
Copenhagen City Heart Study (CCHS) and Copenhagen General Population Study (CGPS) (combined), Denmark	Afzal et al., 2014	35,334	789	HR	Per 20 nmol/L lower	1.16 (0.98-1.38)

Cause of Death<br>Study Name, Location	Author,<br>Publication Year	Number of Participants	Number of Deaths	Estimate	25(OH)D Contrast/Metric	Estimate (95% CI)
Copenhagen vitamin D (CopD) study, Denmark	Durup et al., 2015	243,672	1574	HR	~12.5 versus 75 nmol/L	1.8 (1.6-2.1)
					~125 versus 75 nmol/L	1.2 (1.0-1.5)
ESTHER, Germany	Perna et al., 2013	7709	41	HR	<30 versus ≥50 nmol/L	1.91 (0.77-4.79)
					Per 25 nmol/L increase	0.86 (0.60-1.22)
General Population Trial of Linxian, China	Lin et al., 2012	1101	279	HR	Highest quartile (≥48.4 nmol/L) versus lowest quartile (<19.6 nmol/L)	1.29 (0.92-1.82)
					Per 15 nmol/L increase	1.05 (0.98-1.12)
Mini-Finland Health Survey, Finland	Kilkkinen et al., 2009	6219	293	HR	Highest quintile (men 62.0-180.0, women 56.0-151.0 nmol/L) versus lowest quintile (men 5.0-28.0, women 4.0-25.0 nmol/L)	0.48 (0.31-0.75)
NHANES III, U.S.	Michos et al., 2012	7981	176	HR	<37.5 versus ≥37.5 nmol/L	1.74 (0.94-3.20)
		5001	116		Whites	2.13 (1.01-4.50)
		2980	60		Blacks	0.93 (0.49-1.80)
Whitehall study, UK	Tomson et al., 2013	5409	378	RR	Two-fold higher concentration	0.81 (0.63-1.03)
**Haemorrhagic stroke**						
Mini-Finland Health Survey, Finland	Kilkkinen et al., 2009	6219	43	HR	Highest tertile (men 52.0-180.0, women 48.0-151.0 nmol/L) versus lowest tertile (men 5.0-34.0, women 4.0-31.0 nmol/L)	0.61 (0.26-1.46)
**Ischaemic stroke**						
Mini-Finland Health Survey, Finland	Kilkkinen et al., 2009	6219	175	HR	Highest tertile (men 52.0-180.0, women 48.0-151.0 nmol/L) versus lowest tertile (men 5.0-34.0, women 4.0-31.0 nmol/L)	0.60 (0.38-0.93)

[1] Unadjusted.

HR, hazard ratio; OR, odds ratio; RR, relative risk

Table S4: Results from prospective studies of circulating 25-hydroxyvitamin D and cancer mortality.

Cancer Mortality<br>Study Name, Location	Author,<br>Publication Year	Number of Participants	Number of Deaths	Estimate	25(OH)D Contrast/Metric	Estimate (95% CI)
**Cancer**						
Calcium Intake Fracture Outcome Study (CAIFOS), Australia	Wong et al., 2015	1188	84	HR	<64 versus ≥ 64 nmol/L	1.61 (1.02-2.54)
					<46 versus ≥83 nmol/L	2.63 (1.26-5.45)
					Per 30 nmol/L decrease	1.33 (1.03-1.72)
Copenhagen City Heart Study (CCHS), Denmark	Afzal et al., 2014	9902	2161	HR	Per 20 nmol/L lower	1.14 (1.04-1.25)
Copenhagen General Population Study (CGPS), Denmark	Afzal et al., 2014	25,432	380	HR	Per 20 nmol/L lower	1.00 (0.79-1.26)
European Male Ageing Study (EMAS), Europe	Lee et al., 2014	2452	72	HR	Lowest quartile (<40 nmol/L) versus highest quartile (>79.1 nmol/L)	2.10 (0.94-4.70)
					<25 nmol/L versus ≥75 nmol/L	3.33 (1.38-8.04)
					Excluding those who had ever been treated for cancer	2.55 (1.03-6.33)
					Per SD decrease	1.31 (0.92-1.86)
EPIC-Norfolk, UK	Khaw et al., 2014	14,641	1086	HR	≥90 versus <30 nmol/L	0.90 (0.65-1.26)
					Per 20 nmol/L increase	0.94 (0.89-1.00)
ESTHER, Germany	Schöttker et al., 2013	9554	433	HR	<30 versus >50 nmol/L	1.42 (1.08-1.88)
ESTHER, Germany	Schöttker et al., 2014	9506	498	HR	<30 nmol/L versus ≥50 nmol/L	
					Not time-dependent	1.30 (1.00-1.69)
					Time-dependent	1.25 (0.96-1.62)
ESTHER, Germany	Brenner et al., 2017	9554	629	HR	Per 20 nmol/L increase	0.96 (0.90-1.03)
General Population Trial of Linxian, China	Lin et al., 2012	1101	217	HR	Highest quartile (≥48.4 nmol/L) versus lowest quartile (<19.6 nmol/L)	0.96 (0.66-1.39)
					Per 15 nmol/L increase	0.97 (0.89-1.05)
Health ABC, U.S.	Kritchevsky et al., 2012	2638	218	HR	<30 versus ≥75 nmol/L	1.79 (0.89-3.60)
		1615	117		Whites	4.14 (1.40-12.24)
		1023	101		Blacks	1.60 (0.57-4.48)
MONICA, Switzerland	Rohrmann et al., 2013	3191	188	HR	Highest quartile (62.2-249.1 nmol/L) versus lowest quartile (0-33.5 nmol/L)	0.86 (0.58-1.28)
			72			
			116		Men	0.52 (0.30-0.90)
					Women	1.47 (0.75-2.85)
					Per 25 nmol/L increase	0.92 (0.78-1.07)
					Men	0.72 (0.57-0.91)
					Women	1.14 (0.93-1.39)
Monica10 and Inter99 (combined), Denmark	Skaaby et al., 2012	8329	301	HR	Highest quartile (Monica10, 81-204; Inter99, 65-255 nmol/L) versus lowest quartile (Monica10, 13-45; Inter99, 10-32 nmol/L)	0.81 (0.57-1.2)
Osteoporotic Fractures in Men (MrOS) study, U.S.	Cawthon et al., 2010	1473	97	HR	Lowest quartile (<49.7 nmol/L) versus highest quartile (≥74.9 nmol/L)	0.52 (0.27-1.00)
					Per SD (19.9 nmol/L) decrease	0.80 (0.64-0.99)
NHANES III, U.S.	Freedman et al., 2007	16,818	536	RR	80 to <100 versus <50 nmol/L	1.00 (0.71-1.40)
					≥120 versus <50 nmol/L	1.49 (0.85-2.64)

Cancer Mortality<br>Study Name, Location	Author,<br>Publication Year	Number of Participants	Number of Deaths	Estimate	25(OH)D Contrast/Metric	Estimate (95% CI)
NHANES III, U.S.	Freedman et al., 2010	16,819	884	RR	80 to <100 versus <37.5 nmol/L	1.12 (0.80-1.57)
		7905	513		Men	1.66 (1.06-2.61)
		8914	371		Women	0.86 (0.50-1.46)
					≥100 versus <37.5 nmol/L	1.15 (0.79-1.68)
					Men	1.85 (1.02-3.35)
					Women	0.64 (0.35-1.18)
NHANES III, U.S.	Melamed et al., 2008	13,331	424	Rate ratio	Lowest quartile (<44.4 nmol/L) versus highest quartile (>80.1 nmol/L)	0.91 (0.63-1.31)
NHANES III, U.S.	Liu et al., 2012	13,131	699	HR	<50 versus ≥75 nmol/L	1.37 (1.09-1.71)
NHANES III, U.S.	Sempos et al., 2013	11,315	826	RR	<20 versus 75-99 nmol/L	0.6 (0.3-1.4)
					≥120 versus 75-99 nmol/L	2.1 (0.9-4.8)
NHANES III, U.S.	Schmutz et al., 2016	15,998	844	HR	Highest quartile (>79.9 nmol/L) versus lowest quartile (<44.2 nmol/L)	0.92 (0.66-1.28)
					Per 25 nmol/L increase	1.01 (0.90-1.13)
Rochester Epidemiology Project, U.S.	Dudenkov et al., 2018	11,022	123	HR	<30 versus 50-125 nmol/L	2.56 (1.45-4.53)
					>125 versus 50-125 nmol/L	0.64 (0.20-2.02)
Southern Community Cohort Study (SCCS), U.S.	Signorello et al., 2013	954	477	OR	Lowest quartile (<25.4 nmol/L) versus highest quartile (>54.0 nmol/L)	1.28 (0.78-2.11)
Fourth Tromsø study, Norway	Hutchinson et al., 2010	7161	498	HR	Lowest (mean non-smokers 33.8, smokers 49.2 nmol/L) versus highest quartile (mean non-smokers 72.3, smokers 97.5 nmol/L):	
		4751	273		Non-smokers[b]	1.14 (0.80-1.63)
		2410	225		Smokers[b]	0.82 (0.56-1.21)
Uppsala Longitudinal Study of Adult Men (ULSAM), Sweden	Michaëlsson et al., 2010	1194	164	HR	<5th percentile (<39 nmol/L) versus 10th–90th percentile (46-93 nmol/L)	2.34 (1.36-4.00)
					<10th percentile (<46 nmol/L) versus 10th–90th percentile (46-93 nmol/L)	1.99 (1.29-3.08)
					>90th percentile (>93 nmol/L) versus 10th–90th percentile (46-93 nmol/L)	1.56 (0.95-2.56)
					>95th percentile (>98 nmol/L) versus 10th–90th percentile (46-93 nmol/L)	2.45 (1.36-4.42)
Whitehall study, UK	Tomson et al., 2013	5409	809	RR	Two-fold higher concentration	0.84 (0.71-1.00)
Women's Health Initiative (WHI), U.S.	Eaton et al., 2011	2429	62	HR	Lowest quartile (3.3-36.5) versus highest quartile (65.4-146.7 nmol/L)	1.39 (0.88-2.19)
**Colorectal and anal**						
Copenhagen City Heart Study (CCHS) and Copenhagen General Population Study (CGPS) (combined), Denmark	Afzal et al., 2014	35,334	330	HR	Per 20 nmol/L lower	1.05 (0.84-1.34)
NHANES III, U.S.	Freedman et al., 2007	16,818	66	RR	≥80 versus <50	0.28 (0.11-0.68)
NHANES III, U.S.	Freedman et al., 2010	16,819	95	RR	80 to <100 versus <50 nmol/L	0.61 (0.26-1.47)
					≥100 versus <50 nmol/L	0.35 (0.11-1.14)
NHANES III, U.S.	Fiscella et al., 2011	15,772	91	HR	<50 versus ≥50 nmol/L	2.11 (1.11-4.00)
NHANES III, U.S.	Deng et al., 2013	16,819	86	HR	≥100 versus <50 nmol/L	0.50 (0.15-1.73)
**Gastrointestinal/digestive system**						
Calcium Intake Fracture Outcome Study (CAIFOS), Australia	Wong et al., 2015	1188	27	HR	Per 30 nmol/L decrease	1.08 (0.71-1.61)
Uppsala Longitudinal Study of Adult Men (ULSAM), Sweden	Michaëlsson et al., 2010	1194	NR	HR	<10th percentile (<46 nmol/L) versus 10th–90th percentile (46-93 nmol/L)	3.13 (1.47-6.66)
					>90th percentile (>93 nmol/L) versus 10th–90th percentile (46-93 nmol/L)	2.98 (1.01-8.82)

Cancer Mortality<br>Study Name, Location	Author,<br>Publication Year	Number of Participants	Number of Deaths	Estimate	25(OH)D Contrast/Metric	Estimate (95% CI)
**Upper gastrointestinal**						
General Population Trial of Linxian, China	Lin et al., 2012	1101	175	HR	Highest quartile (≥48.4 nmol/L) versus lowest quartile (<19.6 nmol/L)<br>Per 15 nmol/L increase	0.87 (0.57-1.31)<br>0.97 (0.88-1.06)
**Enterohepatic**						
Uppsala Longitudinal Study of Adult Men (ULSAM), Sweden	Michaëlsson et al., 2010	1194	NR	HR	<10th percentile (<46 nmol/L) versus 10th–90th percentile (46-93 nmol/L)<br>>90th percentile (>93 nmol/L) versus 10th–90th percentile (46-93 nmol/L)	3.11 (1.22-7.89)<br>5.58 (1.77-17.62)
**Pancreatic, liver, and biliary duct**						
Uppsala Longitudinal Study of Adult Men (ULSAM), Sweden	Michaëlsson et al., 2010	1194	NR	HR	<10th percentile (<46 nmol/L) versus 10th–90th percentile (46-93 nmol/L)<br>>90th percentile (>93 nmol/L) versus 10th–90th percentile (46-93 nmol/L)	5.71 (1.81-18.03)<br>10.30 (1.81-58.56)
**Oesophageal, stomach, liver, and pancreatic**						
NHANES III, U.S.	Freedman et al., 2007	16,818	71	RR	≥62.5 versus <62.5	1.42 (0.73-1.76)
NHANES III, U.S.	Freedman et al., 2010	16,819	118	RR	≥80 versus <50 nmol/L	1.31 (0.61-2.81)
**Lung**						
Calcium Intake Fracture Outcome Study (CAIFOS), Australia	Wong et al., 2015	1188	13	HR	Per 30 nmol/L decrease	1.30 (0.70-2.43)
Copenhagen City Heart Study *CCHS) and Copenhagen General Population Study (CGPS) (combined), Denmark	Afzal et al., 2014	35,334	624	HR	Per 20 nmol/L lower	1.28 (1.06-1.54)
NHANES III, U.S.	Freedman et al., 2007	16,818	153	RR	80 to <100 versus <50 nmol/L<br>≥100 versus <50 nmol/L	0.65 (0.36-1.18)<br>1.14 (0.60-2.18)
NHANES III, U.S.	Freedman et al., 2010	16,819	252	RR	80 to <100 versus <50 nmol/L<br>≥100 versus <50 nmol/L	0.99 (0.58-1.70)<br>1.50 (0.90-2.52)
			165		Men:<br>80 to <100 versus <50 nmol/L<br>≥100 versus <50 nmol/L	 1.19 (0.74-1.92)<br>1.87 (1.04-3.34)
			87		Women:<br>≥80 versus <50 nmol/L	 0.64 (0.23-1.79)
NHANES III, U.S.	Cheng et al., 2012	16,693	258	HR	Highest (>80.3 nmol/L) versus lowest quartile (<44.0 nmol/L)	0.95 (0.62-1.44)
**Breast**						
Calcium Intake Fracture Outcome Study (CAIFOS), Australia	Wong et al., 2015	1188	6	HR	Per 30 nmol/L decrease	1.06 (0.43-2.63)
NHANES III, U.S.	Freedman et al., 2007	16,818	28	RR	≥62.5 versus <62.5	0.28 (0.08-0.93)
NHANES III, U.S.	Freedman et al., 2010	16,819	53	RR	≥80 versus <50 nmol/L	0.65 (0.18-2.38)
**Prostate**						
NHANES III, U.S.	Freedman et al., 2007	16,818	47	RR	≥62.5 versus <62.5	0.91 (0.39-2.14)
NHANES III, U.S.	Freedman et al., 2010	16,819	74	RR	≥80 versus <50 nmol/L	1.23 (0.50-3.05)

**Cancer Mortality**<br>**Study Name, Location**	**Author, Publication Year**	**Number of Participants**	**Number of Deaths**	**Estimate**	**25(OH)D Contrast/Metric**	**Estimate (95% CI)**
**Haematological**						
Calcium Intake Fracture Outcome Study (CAIFOS), Australia	Wong et al., 2015	1188	11	HR	Per 30 nmol/L decrease	2.13 (1.00-4.55)
**Non-Hodgkin lymphoma/ leukaemia**						
NHANES III, U.S.	Freedman et al., 2007	16,818	40	RR	≥62.5 versus <62.5	1.34 (0.62-2.91)
NHANES III, U.S.	Freedman et al., 2010	16,819	58	RR	≥80 versus <50 nmol/L	1.10 (0.40-2.98)
**Central nervous system**						
Calcium Intake Fracture Outcome Study (CAIFOS), Australia	Wong et al., 2015	1188	5	HR	Per 30 nmol/L decrease	1.75 (0.57-5.26)
**Miscellaneous (includes cancers of the buccal cavity, larynx, melanoma, gynaecologic sites, kidney, bladder, brain, multiple myeloma, and other cancers)**						
NHANES III, U.S.	Freedman et al., 2007	16,818	130	RR	≥80 versus <50 nmol/L	1.86 (0.75-4.63)
NHANES III, U.S.	Freedman et al., 2010	16,819	234	RR	≥80 versus <50 nmol/L	1.36 (0.71-2.59)

HR, hazard ratio; OR, odds ratio; RR, relative risk

Table S5: Results from prospective studies of circulating 25-hydroxyvitamin D and respiratory disease mortality.

Study Name, Location	Author, Publication Year	Number of Participants	Number of Deaths	Estimate	25(OH)D Contrast/Metric	Estimate (95% CI)
Copenhagen City Heart Study (CCHS) and Copenhagen General Population Study (CGPS) (combined), Denmark	Afzal et al., 2014	35,334	838	HR	Per 20 nmol/L lower	1.51 (1.28-1.77)
EPIC-Norfolk, UK	Khaw et al., 2014	14,641	235	HR	≥90 versus <30 nmol/L	0.24 (0.11-0.54)
					Per 20 nmol/L increase	0.73 (0.63-0.85)
ESTHER, Germany	Schöttker et al., 2013	9554	55	HR	<30 versus >50 nmol/L	2.50 (1.12-5.56)
ESTHER, Germany	Schöttker et al., 2014	9506	64	HR	<30 nmol/L versus ≥50 nmol/L	
					Not time-dependent	2.33 (1.11-4.90)
					Time-dependent	1.86 (0.87-3.98)
Monica10 and Inter99 (combined), Denmark	Skaaby et al., 2012	8329	47	HR	Highest quartile (Monica10, 81-204; Inter99, 65-255 nmol/L) versus lowest quartile (Monica10, 13-45; Inter99, 10-32 nmol/L)	0.26 (0.09-0.75)
Rochester Epidemiology Project, U.S.	Dudenkov et al., 2018	11,022	159	HR	<30 versus 50-125 bnol/L	1.24 (0.59-2.60)
					>125 versus 50-125 nmol/Ll:	1.95 (1.00-3.78)
Whitehall study, UK	Tomson et al., 2013	5409	497	RR	Two-fold higher concentration	0.69 (0.56-0.85)

HR, hazard ratio; RR, relative risk

Table S6: Results from prospective studies of circulating 25-hydroxyvitamin D and other mortality.

Cause of Death<br>Study Name, Location	Author,<br>Publication Year	Number of Participants	Number of Deaths	Estimate	25(OH)D Contrast/Metric	Estimate (95% CI)
**Infectious diseases**						
NHANES III, U.S.	Melamed et al., 2008	13,331	105	Rate ratio	Lowest quartile (<44.4 nmol/L) versus highest quartile (>80.1 nmol/L)	0.84 (0.38-1.86)
**Endocrine, nutritional and metabolic diseases**						
Monica10 and Inter99 (combined), Denmark	Skaaby et al., 2012	8329	15	HR	Highest quartile (Monica10, 81-204; Inter99, 65-255 nmol/L) versus lowest quartile (Monica10, 13-45; Inter99, 10-32 nmol/L)	0.21 (0.04-1.1)
**Diabetes and obesity**						
ESTHER, Germany	Schöttker et al., 2014	9506	31	HR	<30 nmol/L versus ≥50 nmol/L	
					Not time-dependent	6.45 (2.23-18.62)
					Time-dependent	4.95 (1.74-14.12)
**Mental and behavioural disorders**						
Monica10 and Inter99 (combined), Denmark	Skaaby et al., 2012	8329	21	HR	Highest quartile (Monica10, 81-204; Inter99, 65-255 nmol/L) versus lowest quartile (Monica10, 13-45; Inter99, 10-32 nmol/L)	0.44 (0.14-1.4)
**Diseases of the nervous system**						
Monica10 and Inter99 (combined), Denmark	Skaaby et al., 2012	8329	20	HR	Highest quartile (Monica10, 81-204; Inter99, 65-255 nmol/L) versus lowest quartile (Monica10, 13-45; Inter99, 10-32 nmol/L)	0.75 (0.21-2.7)
**Diseases of the nervous system and mental disorders**						
ESTHER, Germany	Schöttker et al., 2014	9506	38	HR	<30 nmol/L versus ≥50 nmol/L	
					Not time-dependent	2.57 (0.99-6.65)
					Time-dependent	2.65 (1.05-6.72)
**Diseases of the digestive system**						
ESTHER, Germany	Schöttker et al., 2014	9506	69	HR	<30 nmol/L versus ≥50 nmol/L	
					Not time-dependent	1.82 (0.89-3.70)
					Time-dependent	2.24 (1.12-4.49)
Monica10 and Inter99 (combined), Denmark	Skaaby et al., 2012	8329	34	HR	Highest quartile (Monica10, 81-204; Inter99, 65-255 nmol/L) versus lowest quartile (Monica10, 13-45; Inter99, 10-32 nmol/L)	0.28 (0.10-0.78)
**Diseases of the genitourinary system**						
ESTHER, Germany	Schöttker et al., 2014	9506	27	HR	<30 nmol/L versus ≥50 nmol/L	
					Not time-dependent	1.99 (0.57-6.95)
					Time-dependent	2.06 (0.56-7.60)
**Infections and not otherwise classified diseases**						
ESTHER, Germany	Schöttker et al., 2014	9506	33	HR	<30 nmol/L versus ≥50 nmol/L	
					Not time-dependent	3.42 (1.34-8.74)
					Time-dependent	3.86 (1.51-9.92)
**Disease death (all-causes excluding deaths due to accidents or suicide**						
Kuopio Ischaemic Heart Disease Risk Factor Study (KIHD), Finland	Mursu et al., 2015	1892	670	HR	Lowest tertile (<32.1 nmol/L) versus highest tertile (>49.4 nmol/L) of 25(OH)D_3	1.31 (1.07-1.60)
**Premature death from all-causes (death at <75 years of age)**						
NHANES III, U.S.	Liu et al., 2012	13,131	1066	HR	<50 versus ≥75 nmol/L	1.40 (1.17-1.68)

Cause of Death<br>Study Name, Location	Author,<br>Publication Year	Number of Participants	Number of Deaths	Estimate	25(OH)D Contrast/Metric	Estimate (95% CI)
**Non-cancer**						
Uppsala Longitudinal Study of Adult Men (ULSAM), Sweden	Michaëlsson et al., 2010	1194	390	HR	<5th percentile (<39 nmol/L) versus 10th–90th percentile (46-93 nmol/L)	1.03 (0.65-1.65)
					<10th percentile (<46 nmol/L) versus 10th–90th percentile (46-93 nmol/L)	1.22 (0.88-1.69)
					>90th percentile (>93 nmol/L) versus 10th–90th percentile (46-93 nmol/L)	1.15 (0.82-1.62)
					>95th percentile (>98 nmol/L) versus 10th–90th percentile (46-93 nmol/L)	1.48 (0.96-2.28)
**Non-cardiovascular**						
MIDSPAN Family Study, Scotland	Welsh et al., 2012	1492	58	HR	<37.5 nmol/L versus ≥37.5 nmol/L	1.82 (0.99-3.35)
NHANES III, U.S.	Ginde et al., 2009	3265	726	HR	<25 versus ≥100 nmol/L	1.42 (0.73-2.79)
Whitehall study, UK	Tomson et al., 2013	5409	1857	RR	Two-fold higher concentration	0.77 (0.69-0.86)
**Non-cancer, non-cardiovascular**						
Copenhagen City Heart Study (CCHS), Denmark	Afzal et al., 2014	9902	1815	HR	Per 20 nmol/L lower	1.23 (1.11-1.37)
Copenhagen General Population Study (CGPS), Denmark	Afzal et al., 2014	25,432	310	HR	Per 20 nmol/L lower	1.38 (1.02-1.86)
Health ABC, U.S.	Kritchevsky et al., 2012	2638	245	HR	<25 versus ≥75 nmol/L	3.01 (1.76-5.13)
		1615	132		Whites	1.29 (0.37-4.52)
		1023	113		Blacks	3.69 (1.77-7.70)
Osteoporotic Fractures in Men (MrOS) study, U.S.	Cawthon et al., 2010	1473	106	HR	Lowest quartile (<49.7 nmol/L) versus highest quartile (≥74.9 nmol/L)	0.94 (0.51-1.72)
					Per SD decrease (19.9 nmol/L)	1.04 (0.83-1.30)
NHANES III, U.S.	Schmutz et al., 2016	15,998	1284	HR	Highest quartile (>79.9 nmol/L) versus lowest quartile (<44.2 nmol/L)	0.67 (0.51-0.88)
					Per 25 nmol/L increase	0.89 (0.82-0.97)
**Non-cancer, non-cardiovascular, non-respiratory**						
EPIC-Norfolk, UK	Khaw et al., 2014	14,641	601	HR	≥90 versus <30 nmol/L	0.82 (0.53-1.28)
					Per 20 nmol/L increase	0.96 (0.88-1.05)
Rochester Epidemiology Project, U.S.	Dudenkov et al., 2018	11,022	316	HR	<30 versus 50-125 bnol/L	2.72 (1.91-3.89)
					>125 versus 50-125 nmol/Ll:	1.12 (0.64-1.97)
Whitehall study, UK	Tomson et al., 2013	5409	551	RR	Two-fold higher concentration	0.78 (0.63-0.95)
**Non-cancer, non-cardiovascular, other non-external causes**						
NHANES III, U.S.	Sempos et al., 2013	11,315	1106	RR	<20 versus 75-99 nmol/L	3.8 (2.3-6.3)
					≥120 versus 75-99 nmol/L	2.0 (0.96-4.0)
Southern Community Cohort Study (SCCS), U.S.	Signorello et al., 2013	1242	621	OR	Lowest quartile (<25.4 nmol/L) versus highest quartile (>54.0 nmol/L)	1.72 (1.15-2.58)
**External causes**						
NHANES III	Melamed et al., 2008	13,331	92	Rate ratio	Lowest quartile (<44.4 nmol/L) versus highest quartile (>80.1 nmol/L)	1.27 (0.56-2.87)

HR, hazard ratio; OR, odds ratio; RR, relative risk

Search strategy

Relevant studies published up to 8 August 2018 were identified through electronic searches using PubMed and EMBASE, without language restriction.

PubMed search:
("vitamin D deficiency"[MeSH Terms] OR "vitamin D deficiency"[Title/Abstract] OR "vitamin D insufficiency"[Title/Abstract] OR "vitamin D status"[Title/Abstract] OR "vitamin D metabolites"[Title/Abstract] OR "25-hydroxyvitamin D"[Title/Abstract] OR "25-hydroxy vitamin D"[Title/Abstract] OR "25(OH)D"[Title/Abstract] OR "25OHD"[Title/Abstract])
AND (mortality [MeSH Terms] OR death [MeSH Terms] OR mortality [Title/Abstract] OR death [Title/Abstract])
AND (cohort studies [MeSH Terms] OR prospective studies [MeSH Terms] OR "follow-up study"[Title/Abstract] OR cohort [Title/Abstract] OR prospective [Title/Abstract] OR prospectively [Title/Abstract] OR observational [Title/Abstract] OR survey [Title/Abstract]
Filters: Humans; Adult: 19+ years

EMBASE search:
("vitamin D deficiency" or "vitamin D insufficiency" or "vitamin D status" or "vitamin D metabolites" or "25-hydroxyvitamin D" or "25-hydroxy vitamin D" or "25(OH)D" or "25OHD"). ti,ab. or "vitamin D deficiency".sh.
AND (mortality or death). ti,ab. or mortality.sh. or death.sh.
AND (cohort or prospective or prospectively or observational or survey or "follow-up study").ti,ab. or "cohort studies".sh. or "prospective studies".sh
Filters: Humans; Adult: 18-64 years and 65+ years

Inclusion criteria:

- Assessed association of measured circulating 25(OH)D with all-cause or cause-specific mortality in adults.
- Participants were recruited from general populations, i.e. participants were not selected on the basis of pre-existing disease.

Exclusion criteria:

- No relevant exposure (no measured circulating 25(OH)D as exposure).
- No relevant outcome (no all-cause mortality or cause-specific mortality as an outcome).
- Review, meta-analysis, Mendelian randomisation study, simulation study, letter, commentary, editorial, conference proceedings, or study protocol.
- Patient population (hospital patients or participants selected on the basis of pre-existing chronic disease such as cardiovascular disease, cancer, diabetes, chronic kidney disease, or any other disease). Studies involving patients referred for coronary angiography were excluded.
- Studies that classified participants based on the presence/absence of a disease or condition such as frailty status, without showing results for all of the participants together.
- Non-adult population.
- Duplicate publications from the same study.
- Insufficient data provided/full-text inaccessible.

References

1. Bolland, M.J.; Bacon, C.J.; Horne, A.M.; Mason, B.H.; Ames, R.W.; Wang, T.K.; Grey, A.B.; Gamble, G.D.; Reid, I.R. Vitamin D insufficiency and health outcomes over 5 y in older women. *Am. J. Clin. Nutr.* **2010**, *91*, 82–89, doi:10.3945/ajcn.2009.28424.
2. Centeno Peláez, V.; Ausín, L.; Ruiz Mambrilla, M.; Gonzalez-Sagrado, M.; Pérez Castrillón, J.L. Severe vitamin D deficiency, functional impairment and mortality in elderly nursing home residents. *J. Aging Res. Clin. Practice* **2014**, *3*, 218–222, doi:10.14283/jarcp.2014.38.
3. Jia, X.; Aucott, L.S.; McNeill, G. Nutritional status and subsequent all-cause mortality in men and women aged 75 years or over living in the community. *Br. J. Nutr.* **2007**, *98*, 593–599, doi:10.1017/s0007114507725163.
4. Kuroda, T.; Shiraki, M.; Tanaka, S.; Ohta, H. Contributions of 25-hydroxyvitamin D, co-morbidities and bone mass to mortality in Japanese postmenopausal women. *Bone* **2009**, *44*, 168–172, doi:10.1016/j.bone.2008.03.023.
5. Pilz, S.; Dobnig, H.; Tomaschitz, A.; Kienreich, K.; Meinitzer, A.; Friedl, C.; Wagner, D.; Piswanger-Solkner, C.; Marz, W.; Fahrleitner-Pammer, A. Low 25-hydroxyvitamin D is associated with increased mortality in female nursing home residents. *J. Clin. Endocrinol. Metab.* **2012**, *97*, E653–657, doi:10.1210/jc.2011-3043.
6. Zhu, K.; Knuiman, M.; Divitini, M.; Hung, J.; Lim, E.M.; Cooke, B.R.; Walsh, J.P. Serum 25-hydroxyvitamin D as a predictor of mortality and cardiovascular events: A 20-year study of a community-based cohort. *Clin. Endocrinol.* **2018**, *88*, 154–163, doi:10.1111/cen.13485.
7. Wong, G.; Lim, W.H.; Lewis, J.; Craig, J.C.; Turner, R.; Zhu, K.; Lim, E.M.; Prince, R. Vitamin D and cancer mortality in elderly women. *BMC Cancer* **2015**, *15*, 106, doi:10.1186/s12885-015-1112-5.
8. Chien, K.L.; Hsu, H.C.; Chen, P.C.; Lin, H.J.; Su, T.C.; Chen, M.F.; Lee, Y.T. Total 25-hydroxyvitamin D concentration as a predictor for all-cause death and cardiovascular event risk among ethnic Chinese adults: a cohort study in a Taiwan community. *PLoS One* **2015**, *10*, e0123097, doi:10.1371/journal.pone.0123097.
9. Brøndum-Jacobsen, P.; Benn, M.; Jensen, G.B.; Nordestgaard, B.G. 25-hydroxyvitamin d levels and risk of ischemic heart disease, myocardial infarction, and early death: population-based study and meta-analyses of 18 and 17 studies. *Arterioscler Thromb. Vasc. Biol.* **2012**, *32*, 2794–2802, doi:10.1161/atvbaha.112.248039.
10. Afzal, S.; Brondum-Jacobsen, P.; Bojesen, S.E.; Nordestgaard, B.G. Genetically low vitamin D concentrations and increased mortality: Mendelian randomisation analysis in three large cohorts. *Br. Med. J.* **2014**, *349*, g6330, doi:10.1136/bmj.g6330.
11. Hirani, V.; Cumming, R.G.; Naganathan, V.; Blyth, F.; Le Couteur, D.G.; Handelsman, D.J.; Waite, L.M.; Seibel, M.J. Associations between serum 25-hydroxyvitamin D concentrations and multiple health conditions, physical performance measures, disability, and all-cause mortality: the Concord Health and Ageing in Men Project. *J. Am. Geriatr. Soc.* **2014**, *62*, 417–425, doi:10.1111/jgs.12693.
12. Deo, R.; Katz, R.; Shlipak, M.G.; Sotoodehnia, N.; Psaty, B.M.; Sarnak, M.J.; Fried, L.F.; Chonchol, M.; de Boer, I.H.; Enquobahrie, D., et al. Vitamin D, parathyroid hormone, and sudden cardiac death: results from the Cardiovascular Health Study. *Hypertension* **2011**, *58*, 1021–1028, doi:10.1161/hypertensionaha.111.179135.
13. Kestenbaum, B.; Katz, R.; de Boer, I.; Hoofnagle, A.; Sarnak, M.J.; Shlipak, M.G.; Jenny, N.S.; Siscovick, D.S. Vitamin D, parathyroid hormone, and cardiovascular events among older adults. *J. Am. Coll. Cardiol.* **2011**, *58*, 1433–1441, doi:10.1016/j.jacc.2011.03.069.
14. de Boer, I.H.; Levin, G.; Robinson-Cohen, C.; Biggs, M.L.; Hoofnagle, A.N.; Siscovick, D.S.; Kestenbaum, B. Serum 25-hydroxyvitamin D concentration and risk for major clinical disease events in a community-based population of older adults: a cohort study. *Ann. Intern. Med.* **2012**, *156*, 627–634, doi:10.7326/0003-4819-156-9-201205010-00004.
15. Saliba, W.; Barnett, O.; Rennert, H.S.; Rennert, G. The risk of all-cause mortality is inversely related to serum 25(OH)D levels. *J. Clin. Endocrinol. Metab.* **2012**, *97*, 2792–2798, doi:10.1210/jc.2012-1747.
16. Saliba, W.; Barnett-Griness, O.; Rennert, G. Obesity and association of serum 25(OH)D levels with all-cause mortality. *Calcif. Tissue Int.* **2014**, *95*, 222–228, doi:10.1007/s00223-014-9885-0.
17. Durup, D.; Jorgensen, H.L.; Christensen, J.; Schwarz, P.; Heegaard, A.M.; Lind, B. A reverse J-shaped association of all-cause mortality with serum 25-hydroxyvitamin D in general practice: the CopD study. *J Clin Endocrinol. Metab.* **2012**, *97*, 2644–2652, doi:10.1210/jc.2012-1176.
18. Durup, D.; Jorgensen, H.L.; Christensen, J.; Tjonneland, A.; Olsen, A.; Halkjaer, J.; Lind, B.; Heegaard, A.M.; Schwarz, P. A reverse J-shaped association between serum 25-hydroxyvitamin D and cardiovascular disease mortality: the CopD study. *J. Clin. Endocrinol. Metab.* **2015**, *100*, 2339–2346, doi:10.1210/jc.2014-4551.

19. Schierbeck, L.L.; Rejnmark, L.; Tofteng, C.L.; Stilgren, L.; Eiken, P.; Mosekilde, L.; Kober, L.; Jensen, J.E. Vitamin D deficiency in postmenopausal, healthy women predicts increased cardiovascular events: a 16-year follow-up study. *Eur. J. Endocrinol.* **2012**, *167*, 553–560, doi:10.1530/eje-12-0283.
20. Lee, D.M.; Vanderschueren, D.; Boonen, S.; O'Neill, T.W.; Pendleton, N.; Pye, S.R.; Ravindrarajah, R.; Gielen, E.; Claessens, F.; Bartfai, G., et al. Association of 25-hydroxyvitamin D, 1,25-dihydroxyvitamin D and parathyroid hormone with mortality among middle-aged and older European men. *Age Ageing* **2014**, *43*, 528–535, doi:10.1093/ageing/aft206.
21. Khaw, K.T.; Luben, R.; Wareham, N. Serum 25-hydroxyvitamin D, mortality, and incident cardiovascular disease, respiratory disease, cancers, and fractures: a 13-y prospective population study. *Am. J. Clin. Nutr.* **2014**, *100*, 1361–1370, doi:10.3945/ajcn.114.086413.
22. Perna, L.; Schöttker, B.; Holleczek, B.; Brenner, H. Serum 25-hydroxyvitamin D and incidence of fatal and nonfatal cardiovascular events: a prospective study with repeated measurements. *J. Clin. Endocrinol. Metab.* **2013**, *98*, 4908–4915, doi:10.1210/jc.2013-2424.
23. Schöttker, B.; Haug, U.; Schomburg, L.; Köhrle, J.; Perna, L.; Müller, H.; Holleczek, B.; Brenner, H. Strong associations of 25-hydroxyvitamin D concentrations with all-cause, cardiovascular, cancer, and respiratory disease mortality in a large cohort study. *Am. J. Clin. Nutr.* **2013**, *97*, 782–793, doi:10.3945/ajcn.112.047712.
24. Schöttker, B.; Saum, K.U.; Perna, L.; Ordóñez-Mena, J.M.; Holleczek, B.; Brenner, H. Is vitamin D deficiency a cause of increased morbidity and mortality at older age or simply an indicator of poor health? *Eur. J. Epidemiol.* **2014**, *29*, 199–210, doi:10.1007/s10654-014-9894-3.
25. Brenner, H.; Jansen, L.; Saum, K.U.; Holleczek, B.; Schöttker, B. Vitamin D supplementation trials aimed at reducing mortality have much higher power when focusing on people with low serum 25-hydroxyvitamin D concentrations. *J. Nutr.* **2017**, *147*, 1325–1333, doi:10.3945/jn.117.250191.
26. Lin, S.W.; Chen, W.; Fan, J.H.; Dawsey, S.M.; Taylor, P.R.; Qiao, Y.L.; Abnet, C.C. Prospective study of serum 25-hydroxyvitamin D concentration and mortality in a Chinese population. *Am. J. Epidemiol.* **2012**, *176*, 1043–1050, doi:10.1093/aje/kws285.
27. Kritchevsky, S.B.; Tooze, J.A.; Neiberg, R.H.; Schwartz, G.G.; Hausman, D.B.; Johnson, M.A.; Bauer, D.C.; Cauley, J.A.; Shea, M.K.; Cawthon, P.M., et al. 25-Hydroxyvitamin D, parathyroid hormone, and mortality in black and white older adults: the health ABC study. *J. Clin. Endocrinol. Metab.* **2012**, *97*, 4156-4165, doi:10.1210/jc.2012-1551.
28. Wong, Y.Y.; McCaul, K.A.; Yeap, B.B.; Hankey, G.J.; Flicker, L. Low vitamin D status is an independent predictor of increased frailty and all-cause mortality in older men: the Health in Men Study. *J. Clin. Endocrinol. Metab.* **2013**, *98*, 3821–3828, doi:10.1210/jc.2013-1702.
29. Pilz, S.; Dobnig, H.; Nijpels, G.; Heine, R.J.; Stehouwer, C.D.A.; Snijder, M.B.; Van Dam, R.M.; Dekker, J.M. Vitamin D and mortality in older men and women. *Clin. Endocrinol.* **2009**, *71*, 666–672, doi:10.1111/j.1365-2265.2009.03548.x.
30. Sun, Y.Q.; Langhammer, A.; Skorpen, F.; Chen, Y.; Mai, X.M. Serum 25-hydroxyvitamin D level, chronic diseases and all-cause mortality in a population-based prospective cohort: the HUNT Study, Norway. *BMJ Open* **2017**, *7*, e017256, doi:10.1136/bmjopen-2017-017256.
31. Semba, R.D.; Houston, D.K.; Bandinelli, S.; Sun, K.; Cherubini, A.; Cappola, A.R.; Guralnik, J.M.; Ferrucci, L. Relationship of 25-hydroxyvitamin D with all-cause and cardiovascular disease mortality in older community-dwelling adults. *Eur. J. Clin. Nutr.* **2010**, *64*, 203-209, doi:10.1038/ejcn.2009.140.
32. Virtanen, J.K.; Nurmi, T.; Voutilainen, S.; Mursu, J.; Tuomainen, T.P. Association of serum 25-hydroxyvitamin D with the risk of death in a general older population in Finland. *Eur. J. Nutr.* **2011**, *50*, 305–312, doi:10.1007/s00394-010-0138-3.
33. Mursu, J.; Nurmi, T.; Voutilainen, S.; Tuomainen, T.P.; Virtanen, J.K. The association between serum 25-hydroxyvitamin D3 concentration and risk of disease death in men: modification by magnesium intake. *Eur. J. Epidemiol.* **2015**, *30*, 343–347, doi:10.1007/s10654-015-0006-9.
34. Vogt, S.; Decke, S.; de Las Heras Gala, T.; Linkohr, B.; Koenig, W.; Ladwig, K.H.; Peters, A.; Thorand, B. Prospective association of vitamin D with frailty status and all-cause mortality in older adults: Results from the KORA-Age Study. *Prev. Med.* **2015**, *73*, 40–46, doi:10.1016/j.ypmed.2015.01.010.
35. Visser, M.; Deeg, D.J.; Puts, M.T.; Seidell, J.C.; Lips, P. Low serum concentrations of 25-hydroxyvitamin D in older persons and the risk of nursing home admission. *Am. J. Clin. Nutr.* **2006**, *84*, 616–622, doi:10.1093/ajcn/84.3.616.

36. El Hilali, J.; de Koning, E.J.; van Ballegooijen, A.J.; Lips, P.; Sohl, E.; van Marwijk, H.W.J.; Visser, M.; van Schoor, N.M. Vitamin D, PTH and the risk of overall and disease-specific mortality: Results of the Longitudinal Aging Study Amsterdam. *J. Steroid. Biochem. Mol. Biol.* **2016**, *164*, 386–394, doi:10.1016/j.jsbmb.2015.12.001.
37. Heath, A.K.; Williamson, E.J.; Kvaskoff, D.; Hodge, A.M.; Ebeling, P.R.; Baglietto, L.; Neale, R.E.; Giles, G.G.; Eyles, D.W.; English, D.R. 25-Hydroxyvitamin D concentration and all-cause mortality: the Melbourne Collaborative Cohort Study. *Public Health Nutr.* **2017**, *20*, 1775–1784, doi:10.1017/s1368980016000501.
38. Welsh, P.; Doolin, O.; McConnachie, A.; Boulton, E.; McNeil, G.; Macdonald, H.; Hardcastle, A.; Hart, C.; Upton, M.; Watt, G., et al. Circulating 25OHD, dietary vitamin D, PTH, and calcium associations with incident cardiovascular disease and mortality: the MIDSPAN Family Study. *J. Clin. Endocrinol. Metab.* **2012**, *97*, 4578–4587, doi:10.1210/jc.2012-2272.
39. Kilkkinen, A.; Knekt, P.; Aro, A.; Rissanen, H.; Marniemi, J.; Heliovaara, M.; Impivaara, O.; Reunanen, A. Vitamin D status and the risk of cardiovascular disease death. *Am. J. Epidemiol.* **2009**, *170*, 1032–1039, doi:10.1093/aje/kwp227.
40. Szulc, P.; Claustrat, B.; Delmas, P.D. Serum concentrations of 17beta-E_2 and 25- hydroxycholecalciferol (25OHD) in relation to all-cause mortality in older men - The MINOS study. *Clin. Endocrinol.* **2009**, *71*, 594–602, doi:10.1111/j.1365-2265.2009.03530.x.
41. Rohrmann, S.; Braun, J.; Bopp, M.; Faeh, D. Inverse association between circulating vitamin D and mortality--dependent on sex and cause of death? *Nutr. Metab. Cardiovasc. Dis.* **2013**, *23*, 960–966, doi:10.1016/j.numecd.2013.05.005.
42. Skaaby, T.; Husemoen, L.L.; Linneberg, A. Does liver damage explain the inverse association between vitamin D status and mortality? *Ann. Epidemiol.* **2013a**, *23*, 812–814, doi:10.1016/j.annepidem.2013.10.002.
43. Skaaby, T.; Husemoen, L.L.; Pisinger, C.; Jorgensen, T.; Thuesen, B.H.; Fenger, M.; Linneberg, A. Vitamin D status and cause-specific mortality: a general population study. *PLoS One* **2012**, *7*, e52423, doi:10.1371/journal.pone.0052423.
44. Skaaby, T.; Husemoen, L.L.; Pisinger, C.; Jorgensen, T.; Thuesen, B.H.; Fenger, M.; Linneberg, A. Vitamin D status and incident cardiovascular disease and all-cause mortality: a general population study. *Endocrine* **2013b**, *43*, 618–625, doi:10.1007/s12020-012-9805-x.
45. Cawthon, P.M.; Parimi, N.; Barrett-Connor, E.; Laughlin, G.A.; Ensrud, K.E.; Hoffman, A.R.; Shikany, J.M.; Cauley, J.A.; Lane, N.E.; Bauer, D.C., et al. Serum 25-hydroxyvitamin D, parathyroid hormone, and mortality in older men. *J. Clin. Endocrinol. Metab.* **2010**, *95*, 4625–4634, doi:10.1210/jc.2010-0638.
46. Johansson, H.; Oden, A.; Kanis, J.; McCloskey, E.; Lorentzon, M.; Ljunggren, O.; Karlsson, M.K.; Thorsby, P.M.; Tivesten, A.; Barrett-Connor, E., et al. Low serum vitamin D is associated with increased mortality in elderly men: MrOS Sweden. *Osteoporos Int.* **2012**, *23*, 991–999, doi:10.1007/s00198-011-1809-5.
47. Bates, C.J.; Hamer, M.; Mishra, G.D. A study of relationships between bone-related vitamins and minerals, related risk markers, and subsequent mortality in older British people: the National Diet and Nutrition Survey of People Aged 65 Years and Over. *Osteoporos Int.* **2012**, *23*, 457–466, doi:10.1007/s00198-011-1543-z.
48. Granic, A.; Aspray, T.; Hill, T.; Davies, K.; Collerton, J.; Martin-Ruiz, C.; von Zglinicki, T.; Kirkwood, T.B.; Mathers, J.C.; Jagger, C. 25-hydroxyvitamin D and increased all-cause mortality in very old women: the Newcastle 85+ study. *J. Intern. Med.* **2015**, *277*, 456–467, doi:10.1111/joim.12273.
49. Freedman, D.M.; Looker, A.C.; Chang, S.C.; Graubard, B.I. Prospective study of serum vitamin D and cancer mortality in the United States. *J. Natl. Cancer Inst.* **2007**, *99*, 1594–1602, doi:10.1093/jnci/djm204.
50. Melamed, M.L.; Michos, E.D.; Post, W.; Astor, B. 25-hydroxyvitamin D levels and the risk of mortality in the general population. *Arch. Intern. Med.* **2008**, *168*, 1629–1637, doi:10.1001/archinte.168.15.1629.
51. Ginde, A.A.; Scragg, R.; Schwartz, R.S.; Camargo, C.A., Jr. Prospective study of serum 25-hydroxyvitamin D level, cardiovascular disease mortality, and all-cause mortality in older U.S. adults. *J. Am. Geriatr. Soc.* **2009**, *57*, 1595–1603, doi:10.1111/j.1532-5415.2009.02359.x.
52. Fiscella, K.; Franks, P. Vitamin D, race, and cardiovascular mortality: findings from a national US sample. *Ann. Fam. Med.* **2010**, *8*, 11–18, doi:10.1370/afm.1035.
53. Liu, L.; Chen, M.; Hankins, S.R.; Nunez, A.E.; Watson, R.A.; Weinstock, P.J.; Newschaffer, C.J.; Eisen, H.J. Serum 25-hydroxyvitamin D concentration and mortality from heart failure and cardiovascular disease, and premature mortality from all-cause in United States adults. *Am. J. Cardiol.* **2012**, *110*, 834–839, doi:10.1016/j.amjcard.2012.05.013.

54. Freedman, D.M.; Looker, A.C.; Abnet, C.C.; Linet, M.S.; Graubard, B.I. Serum 25-hydroxyvitamin D and cancer mortality in the NHANES III study (1988-2006). *Cancer Res.* **2010**, *70*, 8587–8597, doi:10.1158/0008-5472.Can-10-1420.
55. Fiscella, K.; Winters, P.; Tancredi, D.; Hendren, S.; Franks, P. Racial disparity in death from colorectal cancer: does vitamin D deficiency contribute? *Cancer* **2011**, *117*, 1061–1069, doi:10.1002/cncr.25647.
56. Cheng, T.Y.; Neuhouser, M.L. Serum 25-hydroxyvitamin D, vitamin A, and lung cancer mortality in the US population: a potential nutrient-nutrient interaction. *Cancer Causes Control* **2012**, *23*, 1557–1565, doi:10.1007/s10552-012-0033-8.
57. Michos, E.D.; Reis, J.P.; Post, W.S.; Lutsey, P.L.; Gottesman, R.F.; Mosley, T.H.; Sharrett, A.R.; Melamed, M.L. 25-Hydroxyvitamin D deficiency is associated with fatal stroke among whites but not blacks: The NHANES-III linked mortality files. *Nutrition* **2012**, *28*, 367–371, doi:10.1016/j.nut.2011.10.015.
58. Smit, E.; Crespo, C.J.; Michael, Y.; Ramirez-Marrero, F.A.; Brodowicz, G.R.; Bartlett, S.; Andersen, R.E. The effect of vitamin D and frailty on mortality among non-institutionalized US older adults. *Eur. J. Clin. Nutr.* **2012**, *66*, 1024–1028, doi:10.1038/ejcn.2012.67.
59. Deng, X.; Song, Y.; Manson, J.E.; Signorello, L.B.; Zhang, S.M.; Shrubsole, M.J.; Ness, R.M.; Seidner, D.L.; Dai, Q. Magnesium, vitamin D status and mortality: results from US National Health and Nutrition Examination Survey (NHANES) 2001 to 2006 and NHANES III. *BMC Med.* **2013**, *11*, 187, doi:10.1186/1741-7015-11-187.
60. Sempos, C.T.; Durazo-Arvizu, R.A.; Dawson-Hughes, B.; Yetley, E.A.; Looker, A.C.; Schleicher, R.L.; Cao, G.; Burt, V.; Kramer, H.; Bailey, R.L., et al. Is there a reverse J-shaped association between 25-hydroxyvitamin D and all-cause mortality? Results from the U.S. nationally representative NHANES. *J. Clin. Endocrinol. Metab.* **2013**, *98*, 3001–3009, doi:10.1210/jc.2013-1333.
61. Ford, E.S. Lung function, 25-hydroxyvitamin D concentrations and mortality in US adults. *Eur. J. Clin. Nutr.* **2015**, *69*, 572–578, doi:10.1038/ejcn.2014.162.
62. Daraghmeh, A.H.; Bertoia, M.L.; Al-Qadi, M.O.; Abdulbaki, A.M.; Roberts, M.B.; Eaton, C.B. Evidence for the vitamin D hypothesis: The NHANES III extended mortality follow-up. *Atherosclerosis* **2016**, *255*, 96–101, doi:10.1016/j.atherosclerosis.2016.04.007.
63. Donneyong, M.M.; Taylor, K.C.; Kerber, R.A.; Hornung, C.A.; Scragg, R. Is outdoor recreational activity an independent predictor of cardiovascular disease mortality - NHANES III? *Nutr. Metab. Cardiovasc. Dis.* **2016**, *26*, 735–742, doi:10.1016/j.numecd.2016.02.008.
64. Schmutz, E.A.; Zimmermann, M.B.; Rohrmann, S. The inverse association between serum 25-hydroxyvitamin D and mortality may be modified by vitamin A status and use of vitamin A supplements. *Eur. J. Nutr.* **2016**, *55*, 393–402, doi:10.1007/s00394-015-0860-y.
65. Durazo-Arvizu, R.A.; Dawson-Hughes, B.; Kramer, H.; Cao, G.; Merkel, J.; Coates, P.M.; Sempos, C.T. The reverse J-shaped association between serum total 25-hydroxyvitamin D concentration and all-cause mortality: the impact of assay standardization. *Am. J. Epidemiol.* **2017**, *185*, 720–726, doi:10.1093/aje/kww244.
66. Ford, E.S.; Zhao, G.; Tsai, J.; Li, C. Vitamin D and all-cause mortality among adults in USA: findings from the National Health and Nutrition Examination Survey Linked Mortality Study. *Int. J. Epidemiol.* **2011**, *40*, 998–1005, doi:10.1093/ije/dyq264.
67. Amer, M.; Qayyum, R. Relationship between 25-hydroxyvitamin D and all-cause and cardiovascular disease mortality. *Am. J. Med.* **2013**, *126*, 509–514, doi:10.1016/j.amjmed.2012.11.021.
68. Formiga, F.; Ferrer, A.; Megido, M.J.; Boix, L.; Contra, A.; Pujol, R. Low serum vitamin D is not associated with an increase in mortality in oldest old subjects: the Octabaix three-year follow-up study. *Gerontology* **2014**, *60*, 10–15, doi:10.1159/000351024.
69. Buchebner, D.; McGuigan, F.; Gerdhem, P.; Ridderstrale, M.; Akesson, K. Association between hypovitaminosis D in elderly women and long- and short-term mortality-results from the Osteoporotic Prospective Risk Assessment cohort. *J. Am. Geriatr. Soc.* **2016**, *64*, 990–997, doi:10.1111/jgs.14087.
70. Leu Agelii, M.; Lehtinen-Jacks, S.; Zetterberg, H.; Sundh, V.; Bjorkelund, C.; Lissner, L. Low vitamin D status in relation to cardiovascular disease and mortality in Swedish women - Effect of extended follow-up. *Nutr. Metab. Cardiovasc. Dis.* **2017**, *27*, 1143–1151, doi:10.1016/j.numecd.2017.10.013.
71. Masson, S.; Agabiti, N.; Vago, T.; Miceli, M.; Mayer, F.; Letizia, T.; Wienhues-Thelen, U.; Mureddu, G.F.; Davoli, M.; Boccanelli, A., et al. The fibroblast growth factor-23 and Vitamin D emerge as nontraditional risk factors and may affect cardiovascular risk. *J. Intern. Med.* **2015**, *277*, 318–330, doi:10.1111/joim.12232.

72. Trevisan, C.; Veronese, N.; Maggi, S.; Baggio, G.; Toffanello, E.D.; Zambon, S.; Sartori, L.; Musacchio, E.; Perissinotto, E.; Crepaldi, G., et al. Factors influencing transitions between frailty states in elderly adults: the Progetto Veneto Anziani longitudinal study. *J. Am. Geriatr. Soc.* **2017**, *65*, 179–184, doi:10.1111/jgs.14515.
73. Jassal, S.K.; Chonchol, M.; Von Mhlen, D.; Smits, G.; Barrett-Connor, E. Vitamin D, parathyroid hormone, and cardiovascular mortality in older adults: The Rancho Bernardo study. *Am. J. Med.* **2010**, *123*, 1114–1120, doi:10.1016/j.amjmed.2010.07.013.
74. Dudenkov, D.V.; Mara, K.C.; Petterson, T.M.; Maxson, J.A.; Thacher, T.D. Serum 25-hydroxyvitamin D values and risk of all-cause and cause-specific mortality: a population-based cohort study. *Mayo. Clin. Proc.* **2018**, *93*, 721–730, doi:10.1016/j.mayocp.2018.03.006.
75. Signorello, L.B.; Han, X.; Cai, Q.; Cohen, S.S.; Cope, E.L.; Zheng, W.; Blot, W.J. A prospective study of serum 25-hydroxyvitamin d levels and mortality among African Americans and non-African Americans. *Am. J. Epidemiol.* **2013**, *177*, 171–179, doi:10.1093/aje/kws348.
76. Samefors, M.; Ostgren, C.J.; Molstad, S.; Lannering, C.; Midlov, P.; Tengblad, A. Vitamin D deficiency in elderly people in Swedish nursing homes is associated with increased mortality. *Eur. J. Endocrinol.* **2014**, *170*, 667–675, doi:10.1530/eje-13-0855.
77. Tunstall-Pedoe, H.; Woodward, M.; Hughes, M.; Anderson, A.; Kennedy, G.; Belch, J.; Kuulasmaa, K. Prime mover or fellow traveller: 25-hydroxy vitamin D's seasonal variation, cardiovascular disease and death in the Scottish Heart Health Extended Cohort (SHHEC). *Int. J. Epidemiol.* **2015**, *44*, 1602–1612, doi:10.1093/ije/dyv092.
78. Domiciano, D.S.; Machado, L.G.; Lopes, J.B.; Figueiredo, C.P.; Caparbo, V.F.; Oliveira, R.M.; Scazufca, M.; McClung, M.R.; Pereira, R.M. Bone mineral density and parathyroid hormone as independent risk factors for mortality in community-dwelling older adults: a population-based prospective cohort study in Brazil. The Sao Paulo Ageing & Health (SPAH) Study. *J. Bone Miner. Res.* **2016**, *31*, 1146–1157, doi:10.1002/jbmr.2795.
79. Hutchinson, M.S.; Grimnes, G.; Joakimsen, R.M.; Figenschau, Y.; Jorde, R. Low serum 25-hydroxyvitamin D levels are associated with increased all-cause mortality risk in a general population: the Tromsø study. *Eur. J. Endocrinol.* **2010**, *162*, 935–942, doi:10.1530/eje-09-1041.
80. Michaëlsson, K.; Baron, J.A.; Snellman, G.; Gedeborg, R.; Byberg, L.; Sundstrom, J.; Berglund, L.; Arnlov, J.; Hellman, P.; Blomhoff, R., et al. Plasma vitamin D and mortality in older men: a community-based prospective cohort study. *Am. J. Clin. Nutr.* **2010**, *92*, 841–848, doi:10.3945/ajcn.2010.29749.
81. Tomson, J.; Emberson, J.; Hill, M.; Gordon, A.; Armitage, J.; Shipley, M.; Collins, R.; Clarke, R. Vitamin D and risk of death from vascular and non-vascular causes in the Whitehall study and meta-analyses of 12,000 deaths. *Eur. Heart J.* **2013**, *34*, 1365–1374, doi:10.1093/eurheartj/ehs426.
82. Semba, R.D.; Houston, D.K.; Ferrucci, L.; Cappola, A.R.; Sun, K.; Guralnik, J.M.; Fried, L.P. Low serum 25-hydroxyvitamin D concentrations are associated with greater all-cause mortality in older community-dwelling women. *Nutr. Res.* **2009**, *29*, 525–530, doi:10.1016/j.nutres.2009.07.007.
83. Eaton, C.B.; Young, A.; Allison, M.A.; Robinson, J.; Martin, L.W.; Kuller, L.H.; Johnson, K.C.; Curb, J.D.; Van Horn, L.; McTiernan, A., et al. Prospective association of vitamin D concentrations with mortality in postmenopausal women: results from the Women's Health Initiative (WHI). *Am. J. Clin. Nutr.* **2011**, *94*, 1471–1478, doi:10.3945/ajcn.111.017715.
84. Kitamura, K.; Nakamura, K.; Nishiwaki, T.; Ueno, K.; Hasegawa, M. Low body mass index and low serum albumin are predictive factors for short-term mortality in elderly Japanese requiring home care. *Tohoku J. Exp. Med.* **2010**, *221*, 29–34, doi:10.1620/tjem.221.29.

–
